# Fatty acids abrogate the growth-suppressive effects induced by inhibition of cholesterol flux in pancreatic cancer cells

**DOI:** 10.1186/s12935-023-03138-8

**Published:** 2023-11-17

**Authors:** Yuchuan Li, Manoj Amrutkar, Anette Vefferstad Finstadsveen, Knut Tomas Dalen, Caroline S. Verbeke, Ivar P. Gladhaug

**Affiliations:** 1 Department of Hepato-Pancreato-Biliary Surgery, Institute of Clinical Medicine, University of Oslo, Oslo, Norway; 2 Department of Pharmacology, Institute of Clinical Medicine, University of Oslo, Oslo, Norway; 3 Department of Pathology, Oslo University Hospital Rikshospitalet, Oslo, Norway; 4 Department of Nutrition, Institute of Basic Medical Sciences, University of Oslo, Oslo, Norway; 5 Institute of Basic Medical Sciences, The Norwegian Transgenic Center, University of Oslo, Oslo, Norway; 6 Department of Pathology, Institute of Clinical Medicine, University of Oslo, Oslo, Norway; 7 Department of Hepato-Pancreato-Biliary Surgery, Oslo University Hospital Rikshospitalet, Oslo, Norway

**Keywords:** Pancreatic cancer, Lipid flux, Cholesterol, Fatty acids

## Abstract

**Background:**

Despite therapeutic advances, the prognosis of pancreatic ductal adenocarcinoma (PDAC) remains extremely poor. Metabolic reprogramming is increasingly recognized as a key contributor to tumor progression and therapy resistance in PDAC. One of the main metabolic changes essential for tumor growth is altered cholesterol flux. Targeting cholesterol flux appears an attractive therapeutic approach, however, the complex regulation of cholesterol balance in PDAC cells remains poorly understood.

**Methods:**

The lipid content in human pancreatic duct epithelial (HPDE) cells and human PDAC cell lines (BxPC-3, MIA PaCa-2, and PANC-1) was determined. Cells exposed to eight different inhibitors targeting different regulators of lipid flux, in the presence or absence of oleic acid (OA) stimulation were assessed for changes in viability, proliferation, migration, and invasion. Intracellular content and distribution of cholesterol was assessed. Lastly, proteome profiling of PANC-1 exposed to the sterol O-acyltransferase 1 (SOAT1) inhibitor avasimibe, in presence or absence of OA, was performed.

**Results:**

PDAC cells contain more free cholesterol but less cholesteryl esters and lipid droplets than HPDE cells. Exposure to different lipid flux inhibitors increased cell death and suppressed proliferation, with different efficiency in the tested PDAC cell lines. Avasimibe had the strongest ability to suppress proliferation across the three PDAC cell lines. All inhibitors showing cell suppressive effect disturbed intracellular cholesterol flux and increased cholesterol aggregation. OA improved overall cholesterol balance, reduced free cholesterol aggregation, and reversed cell death induced by the inhibitors. Treatment with avasimibe changed the cellular proteome substantially, mainly for proteins related to biosynthesis and metabolism of lipids and fatty acids, apoptosis, and cell adhesion. Most of these changes were restored by OA.

**Conclusions:**

The study reveals that disturbing the cholesterol flux by inhibiting the actions of its key regulators can yield growth suppressive effects on PDAC cells. The presence of fatty acids restores intracellular cholesterol balance and abrogates the alternations induced by cholesterol flux inhibitors. Taken together, targeting cholesterol flux might be an attractive strategy to develop new therapeutics against PDAC. However, the impact of fatty acids in the tumor microenvironment must be taken into consideration.

**Supplementary Information:**

The online version contains supplementary material available at 10.1186/s12935-023-03138-8.

## Background

Pancreatic ductal adenocarcinoma (PDAC) continues to be a highly fatal malignancy [1]. Despite some modest developments in multi-agent cytotoxic therapies in recent years, the prognosis of PDAC remains extremely poor with a 5-years survival rate for all stages combined of about 11% at best [2]. At the time of diagnosis, most patients have advanced disease or metastasis, while only 15–20% of the patients eligible for potentially curative surgery. The incidence and mortality of PDAC are increasing worldwide [3], and PDAC has been estimated to become the third leading cause of cancer-related death in the EU by 2025 [4] and the second leading cause in the USA by 2030 [5]. Hitherto, clinical progress in PDAC treatment has depended heavily on improvements in conventional chemotherapy regimens directed toward the malignant cells in the tumor tissue [6]. Although treatment options involving well-known concepts of immunotherapy [7] and targeted therapies [8, 9] are emerging for PDAC, novel treatment strategies exploiting altered cellular metabolism are now being explored as possible new approaches to the clinical management of PDAC [10–12].

Metabolic reprogramming has recently been recognized as a hallmark of cancer [13]. Multiple studies have revealed that altered lipid metabolism contributes to carcinogenesis and tumor progression in various cancer types [14, 15]. Among those, alterations in cholesterol metabolism and transport (i.e., cholesterol flux) appear to be ubiquitous and important in multiple cancer types, including PDAC [16]. Reprogrammed cholesterol pathways may facilitate cancer progression in multiple ways. Cholesterol is an essential component for membrane synthesis and must be available in excess for the fast growth and proliferation of cancer cells [16]. It is also concentrated in specialized membrane domains known as lipid rafts, and altered raft-cholesterol levels can alter spatial organization and dynamics of cellular membranes and affect regulatory sites for receptor signaling [17]. In addition, cholesterol is required for integrin recycling and focal adhesion disassembly, which is essential for cancer cell migration and invasion [18]. Cholesterol and its metabolic derivatives such as oxysterols, can act as signaling molecules to support cancer cell growth [19] and metastasis [20, 21]. Cholesterol is also involved in epithelial-mesenchymal transition (EMT) and cancer cell drug resistance [22]. Moreover, intermediate products of the mevalonate pathway during cholesterol synthesis, such as farnesyl diphosphate and geranylgeranyl pyrophosphate, can regulate the function of Ras family proteins by prenylation, and hence affect a broad range of cancer cell behaviors [23, 24].

Epidemiological data indicate a close relationship between cholesterol and the occurrence of PDAC. High dietary cholesterol ingestion as well as a recent decrease in serum levels of total cholesterol have been linked to an increased incidence of PDAC [25–27]. Increased free cholesterol (FC) has been reported both in plasma and tumor tissues of PDAC patients compared to healthy individuals, and in cultured PDAC cells compared to non-cancerous pancreatic duct epithelial cells [28, 29]. Cellular cholesterol flux is regulated at multiple levels by complex and coordinated processes involving cholesterol uptake, synthesis, storage, and transport [30]. PDAC is reported to have increased cholesterol uptake [31–33], enhanced cholesterol synthesis [28], elevated cholesteryl ester (CE) storage [34], and decreased cholesterol efflux [35], all contributing to an increased intracellular cholesterol level These observations indicate that disturbed cholesterol balance is a common feature of PDAC. Therefore, it is plausible to assume that alterations in cholesterol balance potentially affect the development and progression of PDAC. To this end, attempts to suppress various cancers, including PDAC, by disturbing cholesterol flux at different levels have been tested in recent years [16]. Studies targeting cholesterol uptake (e.g., LDLR and SR-B1) [31, 33], cholesterol synthesis (e.g., HMGCR and SQLE) [36, 37], and cholesterol esterification (e.g., SOAT1) [38] have all generally been found to have a suppressive effect on PDAC, although some of these observations remain controversial [39]. However, attempts to target CE lipolysis in PDAC has not been reported.

Due to the complexity of cholesterol flux regulation and the pleiotropic functions of cholesterol and its related metabolites in cancers, many details on cholesterol balance in malignant cells remain poorly understood. Furthermore, some of the widely used cholesterol targeting medications, such as statins for dyslipidemia, could potentially affect cholesterol metabolism in cancer cells. In this study, we investigated the lipid profiles of PDAC cells and expression of key enzymes involved in cholesterol homeostasis. We also tested PDAC cell behavior in response to inhibitors disturbing cholesterol flux at different levels, as well as studied the effects of exogenous lipids on PDAC cholesterol balance.

## Methods

### Cell culture

The PDAC cell lines BxPC-3, MIA PaCa-2 and PANC-1 were obtained from American Type Culture Collection (ATCC; Manassas, VA, USA). The human pancreatic duct epithelial cell line H6c7 (HPDE) was obtained from Kerafest Inc. (#ECA001-FP; Boston, MA, USA). PDAC cells were cultured in Dulbecco’s modified Eagle medium (DMEM; GlutaMAX™, #31966047), supplemented with 10% fetal bovine serum (FBS, #10500064), 1X penicillin–streptomycin (#15140122) and 1X amphotericin B (#15290026). HPDE was cultured in Gibco™ keratinocyte serum-free medium supplemented with human recombinant epidermal growth factor and bovine pituitary extract (#17005042). All cell cultures were maintained at 37 °C with 5% CO_2_. All media and supplements were purchased from Thermo Fisher Scientific (Waltham, MA, USA). For experiments, all cells were maintained in DMEM supplemented with 10% or 1% FBS, with or without exposure to drug treatment or lipid stimulation, as indicated in the respective method section below. Cell culture was routinely assessed for mycoplasma using MycoAlert™ Mycoplasma Detection Kit (#LT07-703; Lonza, Basel, Switzerland). Cells lines were authenticated using short tandem repeat (STR) profiling (Eurofins Genomics, Ebersberg, Germany).

### Stimulation with lipids and lipid flux inhibitors

Cells at 60–70% confluence were incubated in DMEM supplemented with 1% FBS, with different combinations of lipids and drugs. Oleic acid (OA; #O1383; Sigma-Aldrich, St Louis, MO, USA) was bound to bovine serum albumin (#A8806; Sigma-Aldrich) in a 2.5:1 ratio, and FC was complexed to methyl-β-cyclodextrin (#332615; Sigma-Aldrich) in a 1:8 ratio before being added to the media at the desired concentration. The following inhibitors for key enzymes regulating lipid flux and cholesterol metabolism were used: HSL/MGLL: CAY10499 (#10007875–10; Cayman Chemicals, Ann Arbor, MI, USA); HSL: BAY599435 (#HY-102056; MedChemExpress, NJ, USA); NCEH1: JW480 (#SML0792; Sigma-Aldrich); HMGCR: simvastatin (#S6196; Sigma-Aldrich); SOAT1: avasimibe (#PZ0190; Sigma-Aldrich); LAL: lalistat 2 (#SML2053; Sigma-Aldrich); DGAT1: PF-04620110 (#PZ0207; Sigma-Aldrich,); and DGAT2: PF-06424439 (#PZ0233; Sigma-Aldrich). The final concentration was 5 µM for avasimibe and simvastatin, and 10 µM for the remaining inhibitors. All inhibitors were dissolved in dimethyl sulfoxide (DMSO, #D5879-M; Sigma-Aldrich) to make stock solutions, which were added to cell culture medium in a 1:1000 ratio. Cells were stimulated with lipids and/or treated with inhibitors for 24 or 48 h, as specified in the respective method sections.

### Staining, imaging, and quantification of lipid droplets (LDs)

Cells were grown on glass coverslips (#HIRS8000120; Hirschmann Laborgeräte, Eberstadt, Germany) for 1–2 days in DMEM supplemented with 10% FBS until 40–50% confluence was reached. Next, cells were incubated with DMEM supplemented with 10% FBS, 1% FBS, or 1% FBS plus OA or /and FC for 48 h. At the end of incubation, cells were fixed with 2% paraformaldehyde in PBS for 20 min at room temperature, washed three times with PBS, and stained for 25 min with 1 µM BODIPY™ 493/503 (#D3922; Invitrogen, Waltham, MA, USA) to visualize LDs, 1 U/mL Phalloidin-CF®568 (#00044; Biotium, Fremont, CA, USA) to visualize the cytoskeleton, and 5 µM Hoechst 33342 (#62249; Thermo Scientific) to visualize the nuclei. After staining, coverslips were washed three times with PBS, mounted to glass slides with ProLong Diamond antifade mountant (#P36965; Invitrogen) and were allowed to be hardened in the dark for 24 h at room temperature. Images were captured using a Zeiss Apotome.2 fluorescence microscope coupled with an oil immersed 40 × objective (Carl Zeiss AG, Germany). The setting for each channel was adjusted individually at the beginning and kept constant throughout the capturing of all images within the same experiment. LD area and number of nuclei were quantified with ImageJ version 1.52 software (National Institutes of Health, Bethesda, MD, USA) from 30–40 images per experimental condition per cell line. The LD content was presented as LD area per nucleus.

### Lipid extraction and thin-layer chromatography (TLC)

TLC was used to separate and identify major lipid classes in HPDE and PDACs. Cells seeded in 10-cm culture dishes in DMEM supplemented with 10% FBS were grown to 70% confluence, washed three times with cold PBS, harvested in 1 mL PBS, and centrifuged at 12000xg for 5 min at 4 °C. Cell pellets were stored at − 20 °C until use. For lipid extraction, cell pellets were homogenized in 0.5 mL PBS with Bioruptor® Plus sonication device (Diagenode SA, Belgium) at 4 °C. Protein concentration was measured with a bicinchoninic acid assay kit (BCA; #23227; Pierce Biotechnology, Rockford, IL, USA). All samples were adjusted with PBS to contain 1 µg/µL protein before extraction. For lipid extraction, homogenates containing 200 µg protein were transferred to glass conical tubes. Two volumes (400 µL) of lipid extraction solvent (chloroform:heptane:methanol = 4:3:2, v/v/v) were added and strongly vortexed for 3 × 15 s, and kept at 4 °C overnight. The next day, extraction tubes were centrifuged at 1500xg for 15 min. The lower organic phases were collected to clean glass test tubes, dried under N_2_ blow (10 min at 37 °C), sealed, and stored at − 20 °C until use.

The extracted lipids were re-dissolved in 40 µL of chloroform:methanol (2:1). The TLC plate (Silica gel 60, Merck) was fully developed in methanol:ethyl acetate (6:4, v/v) to remove impurities, and dried for 6–8 min at 40 °C. Lipid extracts and lipid standard mix (equal weights of TAG, DAG, MAG, PL, FFA, CE, and FC, 10 ng of each) were spotted on the plate, air-dried briefly, and developed in heptane:diethyl ether:acetic acid (55:45:1, v:v:v) until the migration front reached the 80 mm mark line. The plate was subsequently dried for 5 min at 40 °C and dipped in 10% CuSO_4_ × 5H_2_O (w/v) and 8% H3PO4 (v/v) water solution for 60 s. The excess solution was removed by decanting and the plates were air-dried briefly. To visualize CE and FC, the plate was heated on a hot plate for 10 min at 60 °C. To visualize all lipid classes, the plate was heated for additional 7 min at 150 °C with a glass cover. The plate was imaged with a GelDoc Go Imaging system (Bio-Rad, Hercules, CA, USA). The signal density of lipid bands was quantified using ImageJ. Band intensity was normalized to the phospholipids heated to 150 °C from the same sample.

### Immunoblotting

Cells were lysed in RIPA buffer containing a complete proteinase inhibitor cocktail (#11836170001, Roche, Basel, Switzerland) and phosphatase inhibitors (#P0044, Sigma-Aldrich), and homogenized using Bioruptor^®^ Plus sonication device. Lysates were centrifuged at 14000xg for 10 min at 4 °C, and supernatant was collected. Protein concentration was measured by Pierce BCA protein assay kit, and samples were diluted with Laemmli buffer to obtain a final concentration of 1 µg/µL. Proteins were separated on Criterion™ TGX™ 4–15% Mini-PROTEAN® TGX™ Precast Protein Gels (#4561086, Bio-Rad) and transferred to nitrocellulose membranes using semi-dry transfer. Membranes were stained with Ponceau S solution (#P7170; Sigma-Aldrich) to visualize equal protein loading. Membranes were blocked in Tris-buffered saline containing 0.1% Tween-20 (TBS-T) and 5% skimmed milk powder, and incubated with primary antibodies in TBS-T containing 3% BSA overnight at 4 °C. Subsequently, membranes were incubated with appropriate HRP-conjugated secondary antibodies for 1 h at room temperature. The blots were developed with SuperSignal™ Chemiluminescent Substrate (#34577; Thermo Scientific), visualized and photographed with UVP BioImaging Systems Epi Chemi II Darkroom. The band intensity was quantified using ImageJ. Antibody information is provided in Additional file 1: Table S1. The intensity of actin bands was used as loading control.

### Assessment of cell viability, proliferation, and cell density

Cells were grown in clear base 96-well plates for 24–48 h (BxPC-3 = 10000; PANC-1 = 4000; MIA PaCa-2 = 5000 cells seeded per well) to achieve ~ 50% confluence, followed by various stimulations for 48 h. Cell viability was determined by live cell staining with propidium iodide (PI) and Hoechst 33342. PI stains the nuclei of apoptotic/dead cells, and Hoechst stains the nuclei of both viable and dead cells. At the end of stimulation, PI and Hoechst (5 µM each) were added to the culture media and incubated for 20 min at 37 °C before imaging. Images were captured using a Zeiss Apotome.2 fluorescence microscope coupled with a 5 × objective. The percentage of dead cells was determined by the relative number of PI- and Hoechst-positive nuclei counted by ImageJ. Cell proliferation was determined by measuring relative BrdU incorporation into actively proliferating cells using the BrdU Cell Proliferation ELISA kit (ab126556; Abcam, Cambridge, UK) according to the manufacturer’s instructions. BrdU reagent was added to the culture 24 h before the end of stimulation. Relative cell density was analyzed with crystal violet staining, which detects cells adhered to the plate surface at the end of the experiment. In brief, cells were fixed with 2% paraformaldehyde in PBS and then stained with crystal violet solution (#94448; Sigma-Aldrich), containing 20% methanol, for 30 min at room temperature. Cells were washed three times with PBS and air-dried overnight at room temperature. Crystal violet was extracted by incubation with 100 µL per well 0.05 M HCL-50% Ethanol solution for 30 min at room temperature. The absorbance was measured with a spectrometer at 570 nm.

### Cell migration by wound closure assay

Cells seeded in 12-well plates were allowed to grow for 48 h to achieve full confluence, followed by 20 h of serum starvation to halt proliferation. Next, two parallel scratches were made on the cell monolayer in each well. Detached cells were removed with media change. Cells were treated for 1.5 h with 10 µg/mL mitomycin C (#475820, Sigma-Aldrich) dissolved in DMEM to further block proliferation and washed three times with sterile PBS. DMEM supplemented with 1% FBS plus different inhibitors was added1.5 mL per well to start the stimulation. Bright field pictures at 0 h and 24 h of incubation were taken under a Zeiss microscope coupled with a 5 × objective and analyzed with ImageJ. Cell migration distance was calculated from the wound area covered by the migrating cells during 24 h in each field. All pictures taken from the same well were averaged as one data point. Four to eight wells were analyzed in each treatment group.

### Transwell cell invasion assay

Cells at 70% confluence were treated for 1.5 h with 10 µg/mL mitomycin C dissolved in DMEM to block proliferation, then washed three times with sterile PBS. Cells were trypsinized, washed, counted, and then resuspended in DMEM supplemented with 1% FBS. Cells in 100 µL DMEM with 1% FBS were seeded (BxPC-3 = 78000; PANC-1 = 60000; MIA PaCa-2 = 100000 cells per well) in transwell cell culture inserts (#734–1574, Corning, NY, USA) coated with 1 mg/mL Matrigel™ GFR Membrane Matrix (#11553620, Corning). The lower chambers were filled with 600 µL DMEM supplemented with 10% FBS to build a FBS gradient across the insert membrane. DMSO control or different inhibitors were added to the media on both sides. Cells were allowed to invade through the inserts for 48 h, then fixed for 30 min with 2% PFA. Non-migrated cells in the inserts were scraped off. Cells that had invaded through the inserts were stained with 5 µM Hoechst 33342 in PBS, and pictures were taken with a Zeiss microscope coupled with a 5 × objective. Numbers of cell nuclei were quantified with ImageJ.

### Cholesterol content measurement

When 60% confluence was reached, cells grown in 6-well plates were stimulated with different lipids with or without a combination of different enzyme inhibitors for 48 h. Cells were trypsinized and washed three times with cold PBS. Cell pellets were frozen on dry ice and stored at −  20 °C until further analysis. Cell pellets were added to 200–300 µL reaction buffer provided by the kit and homogenized with Bioruptor® Plus sonication device at 4 °C for ten 30 s/30 s on/off cycles. Cellular cholesterol content was measured with Amplex red cholesterol assay kit (#A12216; Thermo Fisher Scientific) following the manufacturer´s instructions. The presence or absence of cholesteryl esterase in the reaction buffer allowed specific measurement of total cholesterol and FC, respectively. The amount of CE was calculated by total cholesterol minus FC. The protein concentration of the cell homogenate was measured with the Pierce BCA protein assay kit. Cellular cholesterol content was normalized to the protein concentration.

### Filipin staining for cholesterol distribution

Cells grown on glass coverslips were stimulated with different lipids, solely or in combination with enzyme inhibitors for 48 h. After stimulation, cells were fixed with 2% paraformaldehyde in PBS and stained with Filipin (#F4767; Sigma-Aldrich) 50 µg/mL in PBS for one hour at room temperature. Cells were washed three times with PBS and mounted with ProLong Diamond antifade mountant before microscopy. Images were taken with a Zeiss Apotome.2 fluorescence microscope coupled with a 20 × objective, in the channel of DAPI (λex = 353 nm; λem = 465 nm).

### Proteomic analysis

*Sample preparation*: PANC-1 cells seeded in 10-cm petri dishes were grown to 70% confluence and treated with DMSO (1:1000), avasimibe (AVA; 5 µM), OA 100 µM or AVA + OA in DMEM supplemented with 1% FBS for 48 h. Cells were washed three times with cold PBS, harvested into 1 mL PBS containing a complete proteinase inhibitor cocktail and phosphatase inhibitors, and centrifuged at 5000xg for 3 min. Cell pellets were collected and stored at − 80 °C until further processing. Protein concentration was determined by BCA assay (Pierce), and for each replicate an equal amount (10 µg) of protein was precipitated on amine beads, as previously described [40]. The precipitated proteins on beads were dissolved in 50 mM ammonium bicarbonate, reduced, alkylated, and digested with trypsin (1:50, enzyme: protein ratio; Promega) at 37 °C overnight. Digested peptides were acidified and loaded onto Evosep C18 tips.

*LC–MS/MS analysis:* LC–MS/MS analysis was carried out using an Evosep LC system (Evosep Biosystems, Odense, Denmark) coupled to the timsTOF fleX mass spectrometer (Bruker Daltonics, Billerica, MA, USA), using a CaptiveSpray nanoelectrospray ion source (Bruker Daltonics). 200 ng of peptide digest was loaded on a capillary C18 Evosep column (15 cm length, 150 μm inner diameter, 1.5 μm particle size, 120; Evosep). Peptides were separated at 50 °C using a 44 min gradient. The timsTOF fleX was operated in PASEF mode. Mass spectra for MS and MS/MS scans were recorded between m/z 100 and 1700. Ion mobility resolution was set to 0.60–1.60 V·s/cm over a ramp time of 100 ms. The data-dependent acquisition was performed using 10 PASEF MS/MS scans per cycle with a near 100% duty cycle. A polygon filter was applied in the m/z and ion mobility space to exclude low m/z, singly charged ions from PASEF precursor selection. An active exclusion time of 0.4 min was applied to precursors that reached 20 000 intensity units. Collisional energy was ramped stepwise as a function of ion mobility.

*Data analysis:* Raw files from LC–MS/MS analysis were submitted to MaxQuant 2.0.3.0 software for protein identification and label-free quantification (LFQ). Parameters were set as follows: Carbamidomethyl (C) was set as a fixed modification and protein N-acetylation and methionine oxidation as variable modifications. First search error window of 20 ppm and the mains search error of 6 ppm were used. Trypsin without proline restriction enzyme option was used, with two allowed miscleavages. Minimal unique peptides were set to one, and FDR allowed was 0.01 (1%) for peptide and protein identification. The UniProt human database was used. Generation of reversed sequences was selected to assign FDR rate. The proteome data was further processed using Perseus version 1.6.1.3. LFQ intensities were normalized and log_10_ transformed. Qlucore Omics Explorer version 3.8 (Qlucore AB, Lund, Sweden) was used for data visualization and exploration, principal component analysis (PCA), and analysis of differential proteome profiles (Heatmap). In addition, the list of differentially expressed proteins (DEPs) was subjected to the Kyoto Encyclopedia of Genes and Genomes (KEGG) database for pathway analysis, while Gene Ontology (GO) analysis was conducted using the DAVID Bioinformatics Database version 6.8 [41, 42].

### Statistical analysis

Data analysis was performed using GraphPad Prism version 9 (GraphPad Software, CA, USA) or a two-tailed Student’s t-test. *p* < 0.05 was considered statistically significant. Data are presented as means ± SD or means ± 95% confidence interval.

## Results

### Major lipid components, LD storage, and related protein expression in HPDE and PDAC cells

**Fig. 1:**
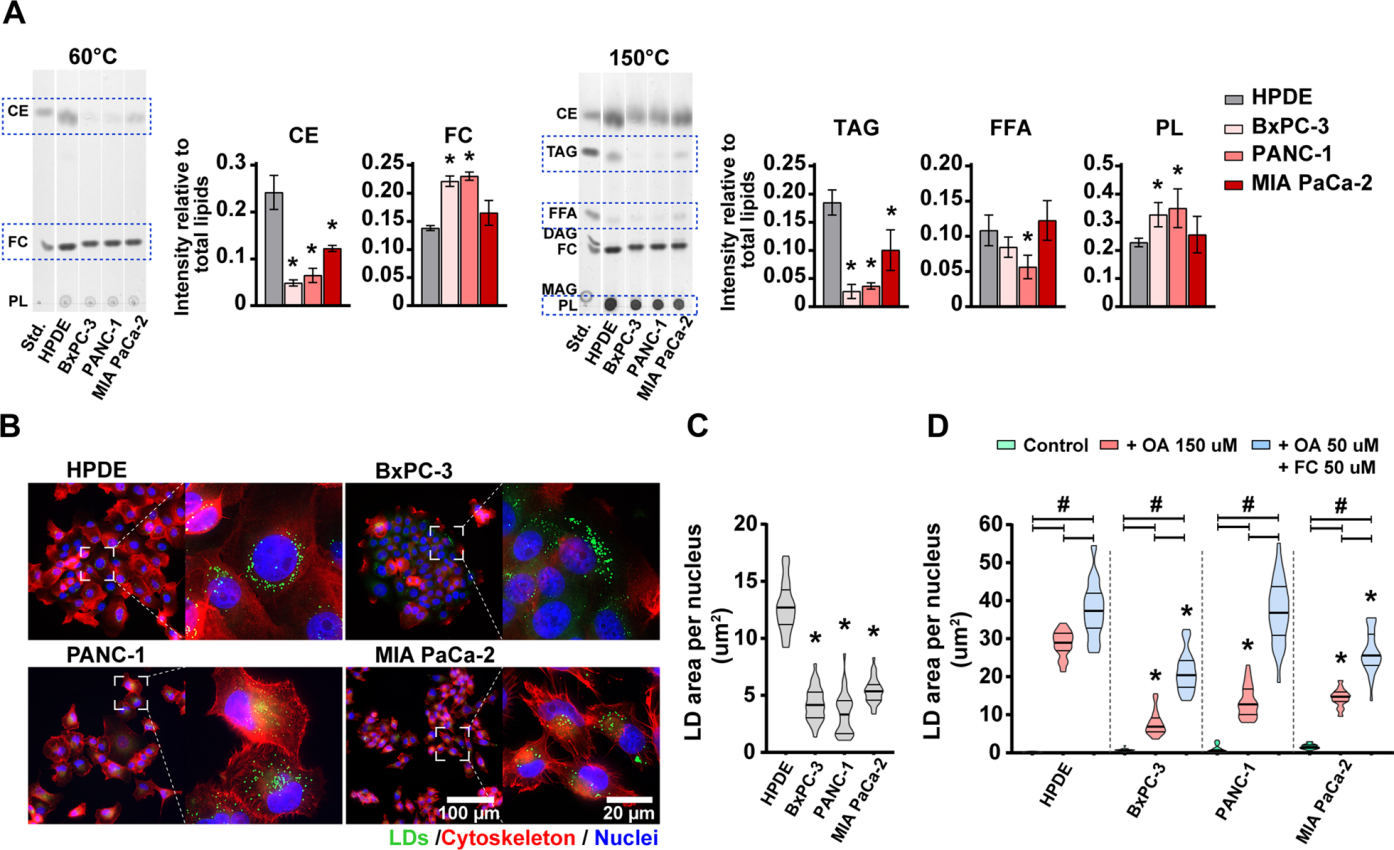
Major lipid classes and LD storage in HPDE and PDAC cells. **A**. TLC images showing major lipid classes in HPDE and PDAC cells cultured in DMEM supplemented with 10% FBS. Band intensity was quantified relative to total lipids of the same sample. Lipid standard mixture (Std.) contains 1 µg for each lipid; **B**. LD storage in HPDE and PDAC cells cultured in DMEM supplemented with 10% FBS. Cells were stained with BODIPY 493/503 to visualize LDs (green), Phallodin-CF568 to visualize cytoskeleton (red), and Hoechst 33,342 to visualize nuclei (blue); **C**-**D**. Quantification of LD content in HPDE and PDAC cells cultured in DMEM, supplemented with 10% or 1% FBS, or 1% FBS plus OA (150 µM), or OA (50 µM) and FC (50 µM). Results are presented as means ± SD (n = 3) in A and means ± 95% confidence interval (n = 30–35 images) in C and D. *p < 0.05 comparing PDAC cells with HPDE cells; ^#^*p* < 0.05 comparing control with OA or OA + FC. *CE* cholesteryl ester, *DAG* diacylglycerol, *FBS* fetal bovine serum, *FC* free cholesterol, *FFA* free fatty acids, *LD* lipid droplet, *MAG* monoacylglycerol, *OA* oleic acid, *PL* phospholipids, *TAG* triacylglycerol, *TLC* thin-layer chromatography

To investigate whether lipid metabolism is altered in pancreatic cancer cells compared to pancreatic duct epithelial cells, we analyzed the major lipid components in HPDE cells and three different PDAC cell lines (BxPC-3, PANC-1 and MIA PaCa-2) by TLC (Fig. 1A). Five major lipid components, CE, FC, triacylglycerol (TAG), free fatty acids (FFA), and phospholipids (PL), were detected in all cell lines, while diacylglycerol (DAG) and monoacylglycerol (MAG) were detected only in trace amount. Both HPDE and PDAC cells contained relatively more CE than TAG. Compared to HPDE cells, all three PDAC cell lines contained less CE and TAG, whereas BxPC-3 and PANC-1 contained more FC and PL. Except for PANC-1, FFA content was not significantly different between PDAC cells and HPDE cells. Among the three PDAC cell lines investigated, BxPC-3 and PANC-1 displayed a similar pattern of the levels of different lipid species measured (Fig. 1A), whereas MIA PaCa-2 showed a pattern contrary to that of the two others.

Since intracellular CE and TAG are mainly stored in LDs, we next analyzed the content of LDs in these cells. Under basal culture conditions (i.e., supplemented with 10% FBS), both HPDE and PDAC cells contained LDs that were clearly visible by BODIPY 493/503 staining (Fig. 1B). In general, PDAC cells contained less LDs than HPDE cells (Fig. 1C), which is consistent with their lower levels of CE and TAG content, as detected by TLC (Fig. 1A). Because exogenous lipids can alter lipid flux and LD storage, we next stimulated the cells with different lipid supplements and analyzed their LD content (Fig. 1D). Cells cultured with reduced serum condition (1% FBS), where exogenous lipids are low, the LD content was nearly absent (Fig. 1D, control group), indicating that exogenous lipids in FBS contribute significantly to both lipid flux and LD storage in HPDE and PDAC cells. When supplemented with OA (150 µM) alone (enough to synthesis 50 µM TAG in theory), or OA (50 µM) combined with FC (50 µM) (enough to synthesis 50 µM CE in theory), the LD content increased substantially in all cell lines (Fig. 1D). Interestingly, all cells stored more LDs in the presence of cholesterol plus OA supplementation than supplemented with OA alone, indicating LDs in these cells were more affected by cellular cholesterol flux than FA flux (Fig. 1D).

Next, we compared expression levels of key proteins involved in LD turnover and cholesterol metabolism (Additional file 2: Fig. S1A) in HPDE and PDAC cells. The expression levels of various lipases (Additional file 2: Fig. S1B), esterification enzymes and LD-associated proteins (Additional file 2: Fig. S1C) involved in the turnover of lipids and LDs was quite heterogeneous, also among the three PDAC cell lines. PDAC cells in general expressed lower SOAT1 (Additional file 2: Fig. S1C) but higher LDLR, HMGCR and SQLE (Additional file 2: Fig. S1D) compared to HPDE, all these enzymes are involved in the regulation of cholesterol flux.

### Inhibition of lipid flux promotes cell death and suppresses proliferation of PDAC cells

**Fig. 2:**
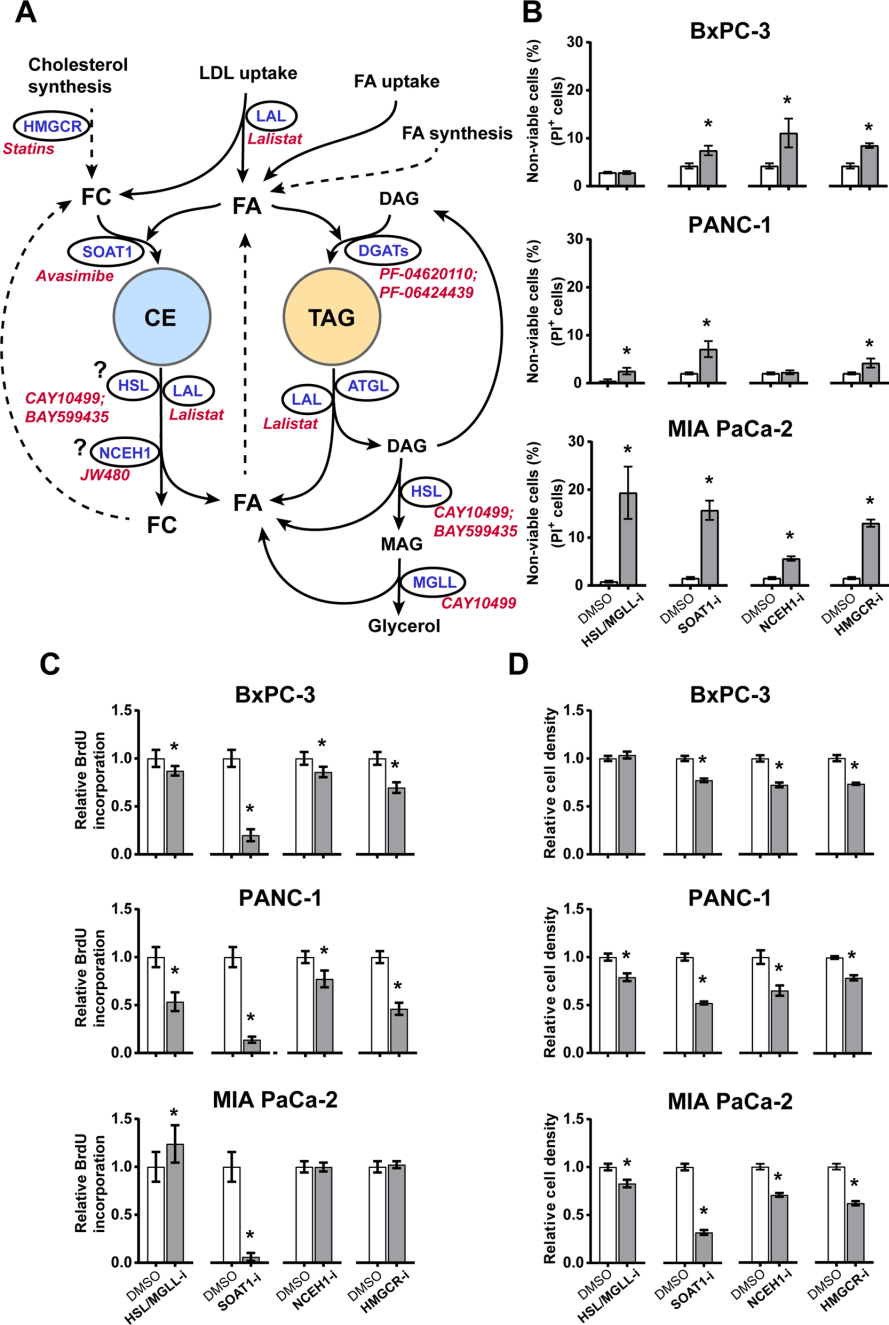
Effect of selected lipid flux inhibitors on PDAC cell viability, proliferation, and cell density. **A**. A schematic presentation of lipid turnover through LDs, the key enzymes, and corresponding inhibitors. **B-D**. PDAC cells were incubated in DMEM supplemented with 1% FBS and various inhibitors (10 µM for HSL/MGLL-i and NCEH1-i, 5 µM for SOAT1-i and HMGCR-i) for 48 h. **B**. Cell viability assessed by the percentage of propidium iodide (PI) positive (non-viable) cells relative to Hoechst 33342 positive (total) cells. **C**. Cell proliferation assessed by BrdU incorporation. **D**. Relative cell density assessed by crystal violet staining. Results are presented as means ± SD (n = 4–5, *p < 0.05 comparing inhibitors with DMSO). *ATGL* adipose triacylglycerol lipase, *CE* cholesteryl ester, *DAG* diacylglycerol, *DGATs* diacylglycerol O-Acyltransferases, *FC* free cholesterol, *FFA* free fatty acids, *HMGCR* 3-Hydroxy-3-Methylglutaryl-CoA reductase, *HSL* hormone-sensitive lipase, *LAL* lysosomal acid lipase, *LD* lipid droplet, *MAG* monoacylglycerol, *MGLL* monoacylglycerol lipase, *NCEH1* neutral cholesterol ester hydrolase 1, *SOAT1* sterol O-acyltransferase 1, *TAG* triacylglycerol

Results shown in Fig. 1 indicate a re-programmed cholesterol metabolism in PDAC cells, which is largely reflected by changes in FC and CE-LD storage as well as in the expression of enzymes associated with cholesterol metabolism. We further wanted to know whether disturbing cholesterol flux and LD storage (both CE and TAG) affects PDAC cell survival/proliferation. To investigate whether alternation in lipid flux affects cell behavior in pancreatic cancer, PDAC cell were treated with a series of small molecule inhibitors that target key enzymes of cellular lipid flux (Fig. 2A). To avoid the impact of excessive exogenous impact of lipids from FBS, the concentration of FBS was reduced to 1% in the subsequent experiments. The impact on cell viability was determined using PI-Hoechst staining (Fig. 2B; Additional file 2: Fig. S2). HSL/MGLL inhibition induced about 20% cell death in MIA PaCa-2, while the same dose was less harmful to PANC-1 and BxPC-3 (Fig. 2B). Inhibition of SOAT1 or of HMGCR increased cell death in all three PDAC cell lines, while being more effective in MIA PaCa-2 cells than BxPC-3 and PANC-1. NCEH1 inhibition induced significant cell death in BxPC-3 and MIA PaCa-2, while not affecting PANC-1. Cell proliferation was assessed by the incorporation of BrdU. HSL/MGLL inhibition reduced cell proliferation most effectively in PANC-1 (Fig. 2C). Among all the inhibitors tested, SOAT1 inhibitor was most effective and largely reduced proliferation of all three PDAC cell lines. Both NCEH1 inhibition and HMGCR inhibition reduced proliferation of BxPC-3 and PANC-1, but had no impact on MIA PaCa-2. Relative cell density measured by crystal violet staining was used to determine the combined effects of these inhibitors on cell death and proliferation. Inhibition of HSL/MGLL reduced cell density of MIA PaCa-2 and PANC-1, but not BxPC-3. Inhibition of SOAT1, NCEH1 or HMGCR reduced cell density in all three PDAC cell lines (Fig. 2D). Overall, Inhibition of HSL/MGLL, SOAT1, NCEH1, or HMGCR displayed a prominent inhibitory effect on PDAC cell growth. In contrast, inhibition of LAL by lalistat 2, DGATs by PF-04620110 and PF-06424439, and selective inhibition of HSL by BAY599435, had no clear impact on PDAC cell density (Additional file 2: Fig. S3).

### Disturbing lipid flux affects PDAC cell migration and invasion

**Fig. 3:**
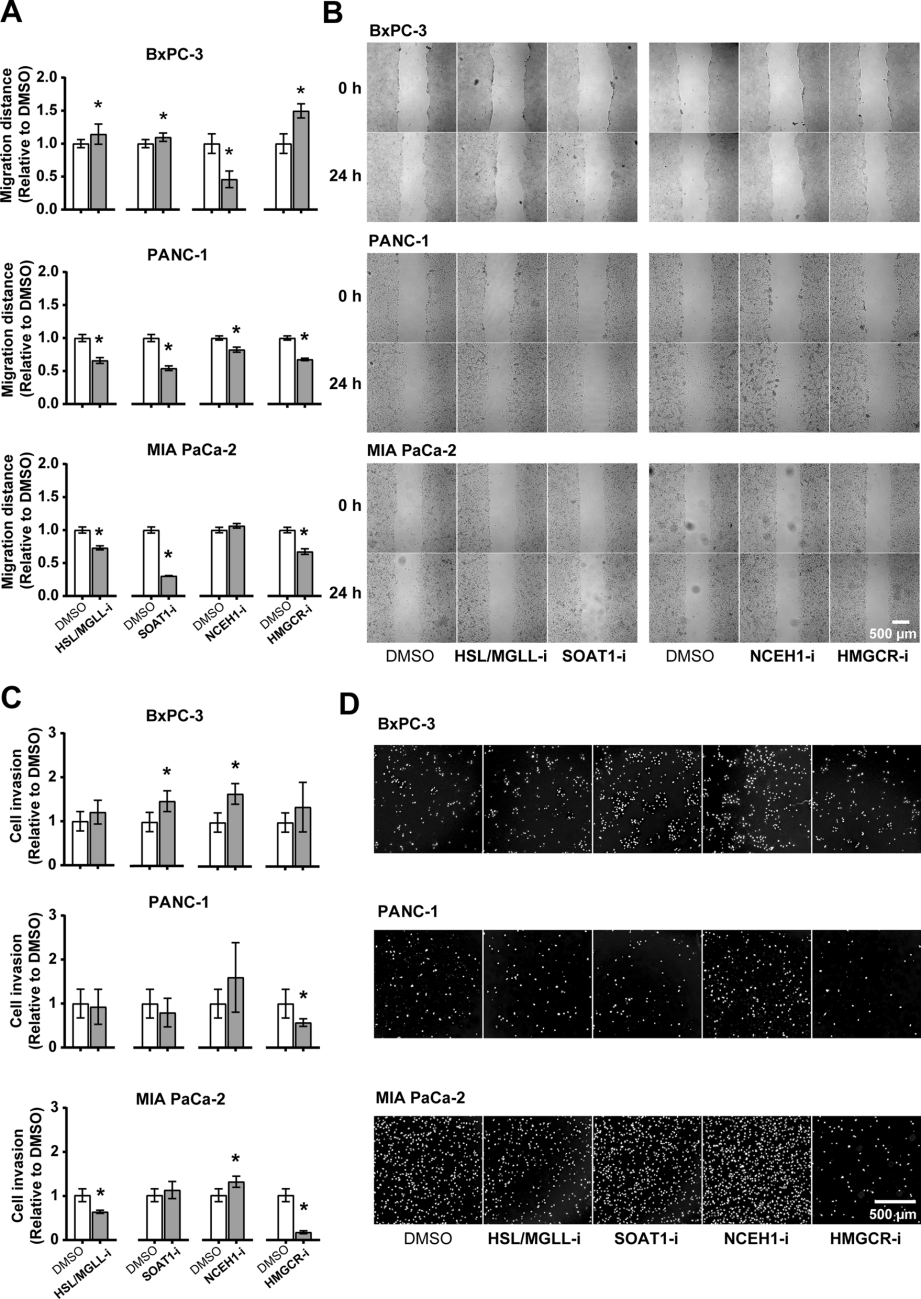
Effect of selected lipid flux inhibitors on PDAC cell migration and invasion. Cells pre-treated with mitomycin C were investigated for **A-B**. migration and **C-D**. invasion following their exposure to lipid flux inhibitors (10 µM for HSL/MGLL-i and NCEH1-i, 5 µM for SOAT1-i and HMGCR-i). **A**. Relative migration distance in 24 h, calculated from the wound-healing assay of confluent cells. **B**. Representative images for the results in A. **C**. Cells invaded through Matrigel at 48 h were stained with Hoechst 33342, photographed and quantified. **D**. Representative images for the results in A. Results are presented as means ± SD (n = 4–8 in A and n = 4 in C, *p < 0.05 comparing inhibitors with DMSO)

We further studied the effect of lipid flux disturbance on PDAC cell migration and invasion with a wound-healing assay and transwell assay, respectively (Fig. 3). The three PDAC cell lines respond differently in terms of cell migration and invasion when exposed to different lipid flux inhibitors. Cell migration of BxPC-3 was suppressed by inhibition of NCEH1, but promoted by inhibition of HSL/MGLL, SOAT1, or HMGCR. Cell migration of MIA PaCa-2 was unaffected by NCEH1 inhibitor, but suppressed by all other inhibitors, while migration of PANC-1 was suppressed by all four inhibitors. Interestingly, HMGCR inhibition prominently increased migration of BxPC-3, but suppressed migration of MIA PaCa-2 and PANC-1 (Fig. 3A, B). inhibition of HSL/MGLL or HMGCR suppressed the invasion of MIA PaCa-2 and PANC-1 (HMGCR inhibition only), without obvious effect on BxPC-3, whereas inhibition of SOAT1 or NCEH1 promoted invasion of BxPC-3 and MIA PaCa-2 (NCEH1 inhibition only), without significantly affecting PANC-1. Notably, inhibition of HMGCR prominently suppressed invasion of MIA PaCa-2 (Fig. 3C, D).

### The effect of exogenous free cholesterol (FC) and fatty acids (FAs)

**Fig. 4:**
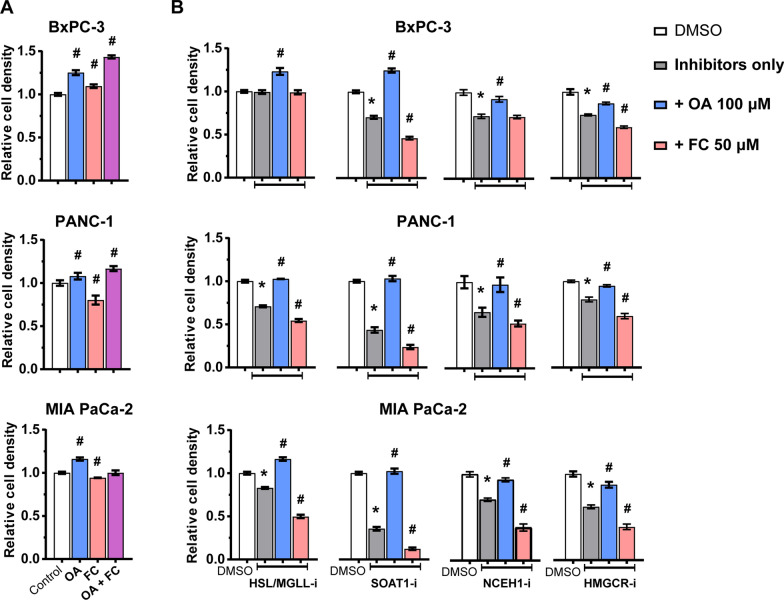
Effect of lipid flux inhibitors, free cholesterol, and fatty acids on PDAC cell density. Cells incubated for 48 h in DMEM supplemented with 1% FBS, with or without OA (100 µM), FC (50 µM) and lipid flux inhibitors (10 µM for HSL/MGLL-i and NCEH1-i, 5 µM for SOAT1-i and HMGCR-i), were assessed for cell density using crystal violet staining. **A**. Effect of OA and FC on PDAC cell density. **B**. Combined effect of lipid flux inhibitors and lipids on PDAC cell density. Results are presented as means ± SD (n = 4–5, *p < 0.05 comparing inhibitors with DMSO; ^#^*p* < 0.05 comparing OA or FC treatment with no lipid treatment). *FC* free cholesterol, *OA* oleic acid

When testing the effect of the enzyme inhibitors CAY10499, avasimibe and JW480, we noticed that their cell suppressive effect was masked by the presence of high FBS (Additional file 2: Fig. S4). To investigate whether lipid components in FBS counteract the effects of the inhibitors, we examined the impact of FA and FC on PDAC cell density (Fig. 4A). OA slightly increased cell density in all three PDAC cell lines. FC had a small but significant effect on BxPC-3 and MIA PaCa-2, but slightly decreased cell density of PANC-1. The combination of OA and FC increased cell density mainly in BxPC-3 and PANC-1, while less prominently in MIA PaCa-2. Next, we studied the combined effect of lipids and lipid flux inhibitors. Surprisingly, OA and FC showed opposite effects in all three PDAC cell lines treated with the various lipid flux inhibitors. A consistent pattern emerged, where the suppressive effects of these inhibitors were enhanced by FC but reversed by OA coincubation (Fig. 4B). It seems like fatty acid excess renders the PDAC cells less sensitive to manipulation of intracellular cholesterol flux.

### Fatty acids promote cellular cholesterol balance in PDAC cells

**Fig. 5:**
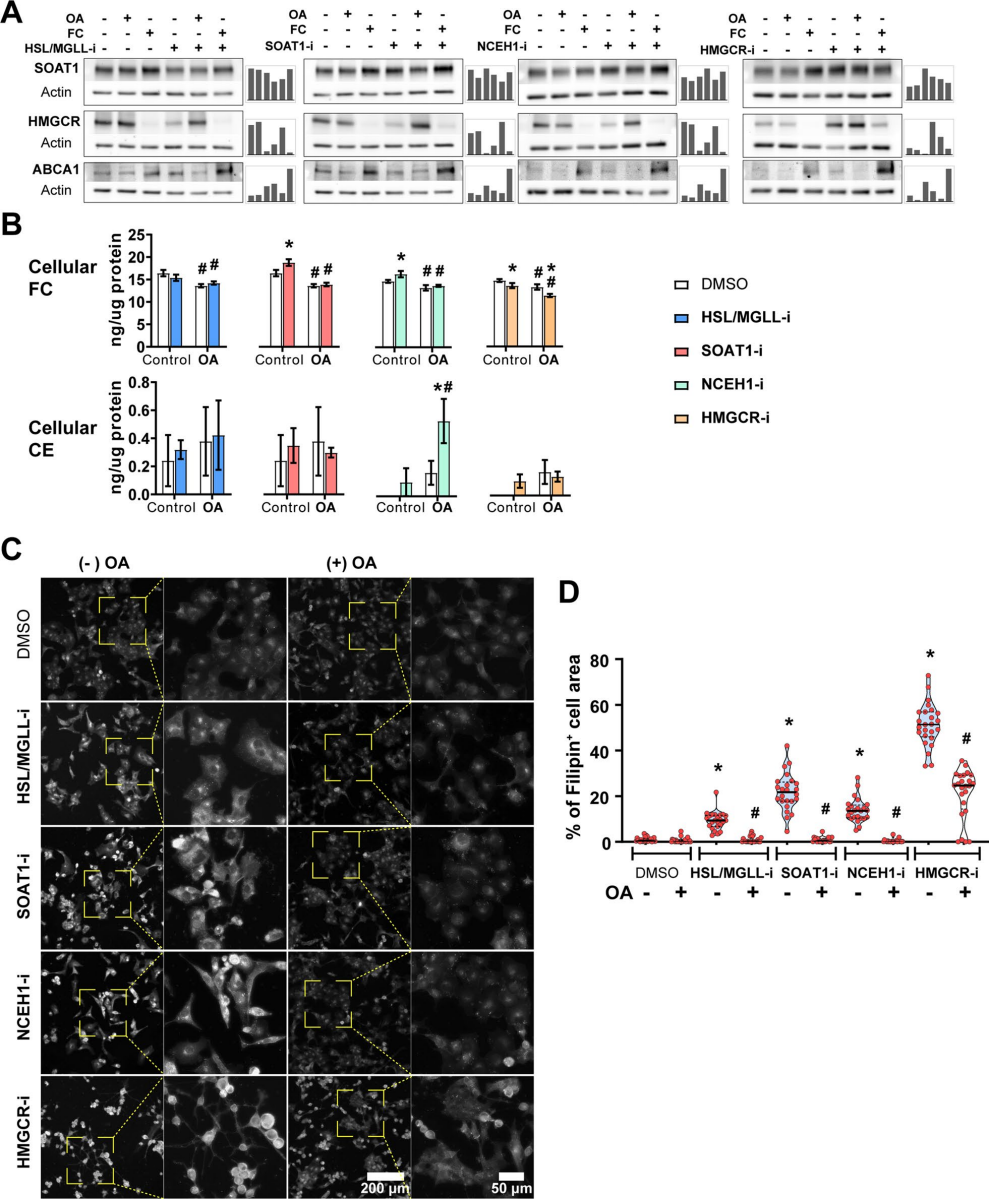
Effect of selected lipid flux inhibitors and oleic acid on cholesterol balance in PANC-1 cells. Cells were incubated for 48 h in DMEM supplemented with 1% FBS, with or without the presence of OA (100 µM), FC (50 µM) and various enzyme inhibitors (10 µM for HSL/MGLL-i and NCEH1-i, 5 µM for SOAT1-i and HMGCR-i). **A**. Expression of key proteins involved in cellular cholesterol storage, synthesis and efflux. **B**. Cellular content of FC and CE. **C**. Representative images of cells stained with Filipin, indicating cellular distribution of FC. **D**. Quantification of Filipin-positive cell area in **C**. Results are presented as means ± SD in **B** (n = 4) and means ± 95% confidence interval in **D** (n = 20–25 images). *p < 0.05 comparing inhibitors with DMSO; ^#^*p* < 0.05 comparing OA treatment with no OA treatment). *CE* cholesteryl esters, *FC* free cholesterol, *OA* oleic acid

The three PDAC cell lines studied responded similarly to lipid flux inhibitors in terms of survival, proliferation and density (Fig. 2B-D). Since PANC-1 expresses considerable levels of all relevant enzymes examined in this study, and it carries an activating mutation of KRAS, which is present in ~ 90% of clinical PDAC, subsequent experiments were performed mainly on PANC-1 cells. We first incubated PANC-1 cells with selected inhibitors, in the presence or absence of OA or FC, and analyzed expression levels of key proteins involved in cholesterol balance (Fig. 5A). SOAT1 is responsible for esterification of FC and subsequent storage as CE in LDs. SOAT1 expression was increased by FC and by inhibition of NCEH1 or HMGCR, was slightly reduced upon inhibition of HSL/MGLL, and was not affected by OA. HMGCR is a key enzyme for cholesterol synthesis. As expected, exogenous FC decreased HMGCR expression, most likely due to feedback inhibition of cholesterol synthesis [43]. Interestingly, inhibition of HSL/MGLL, SOAT1 or NCEH1 also reduced HMGCR expression, while inhibition of HMGCR activity increased its protein expression. The inhibitor-mediated downregulation of HMGCR was restored by OA and enhanced by FC. ABCA1 is responsible for reverse cholesterol transport when cellular cholesterol exceeds a threshold. Expression levels of ABCA1 were increased by FC and further enhanced with inhibitors of HSL/MGLL, SOAT1, NCEH1 and HMGCR, indicating an increased cellular cholesterol burden.

Next, we measured the cellular content of FC and CE (Fig. 5B). In low serum conditions, PANC-1 cells contain mainly FC, with nearly undetectable levels of CE. Inhibition of HSL/MGLL had no clear effect on cholesterol content. Inhibition of SOAT1 or NCEH1 slightly increased FC, while inhibition of HMGCR slightly reduced FC. The presence of OA-induced a small but consistent decrease in FC, independent of the presence of inhibitors. However, inhibition of NCEH1 induced a significant increase in CE content in the presence of OA, indicating that NCEH1 could be an important enzyme for CE degradation in PANC-1 cells. Since we observed only minor alternations in total cellular cholesterol levels, we further investigated the intracellular distribution of FC by Filipin staining (Fig. 5C). The quantification of Filipin signal-enriched area revealed an increased intracellular aggregation of FC across all four lipid flux inhibitors, which was also reversed by OA (Fig. 5C, D).

To investigate whether the observed changes in PANC-1 cells were KRAS dependent, we also examined the effect of fatty acid on cholesterol balance in BxPC-3 cells (Additional file 2: Fig. S5). Interestingly, we observed similar expression pattern of HMGCR and ABCA1 (Additional file 2: Fig. S5A) and intracellular cholesterol distribution (Supplementary Fig. S5B-C) in BxPC-3 cells when compared to PANC-1 cells.

### Inhibition of SOAT1 by avasimibe induces significant changes in total proteome, which are restored by oleic acid

**Fig. 6:**
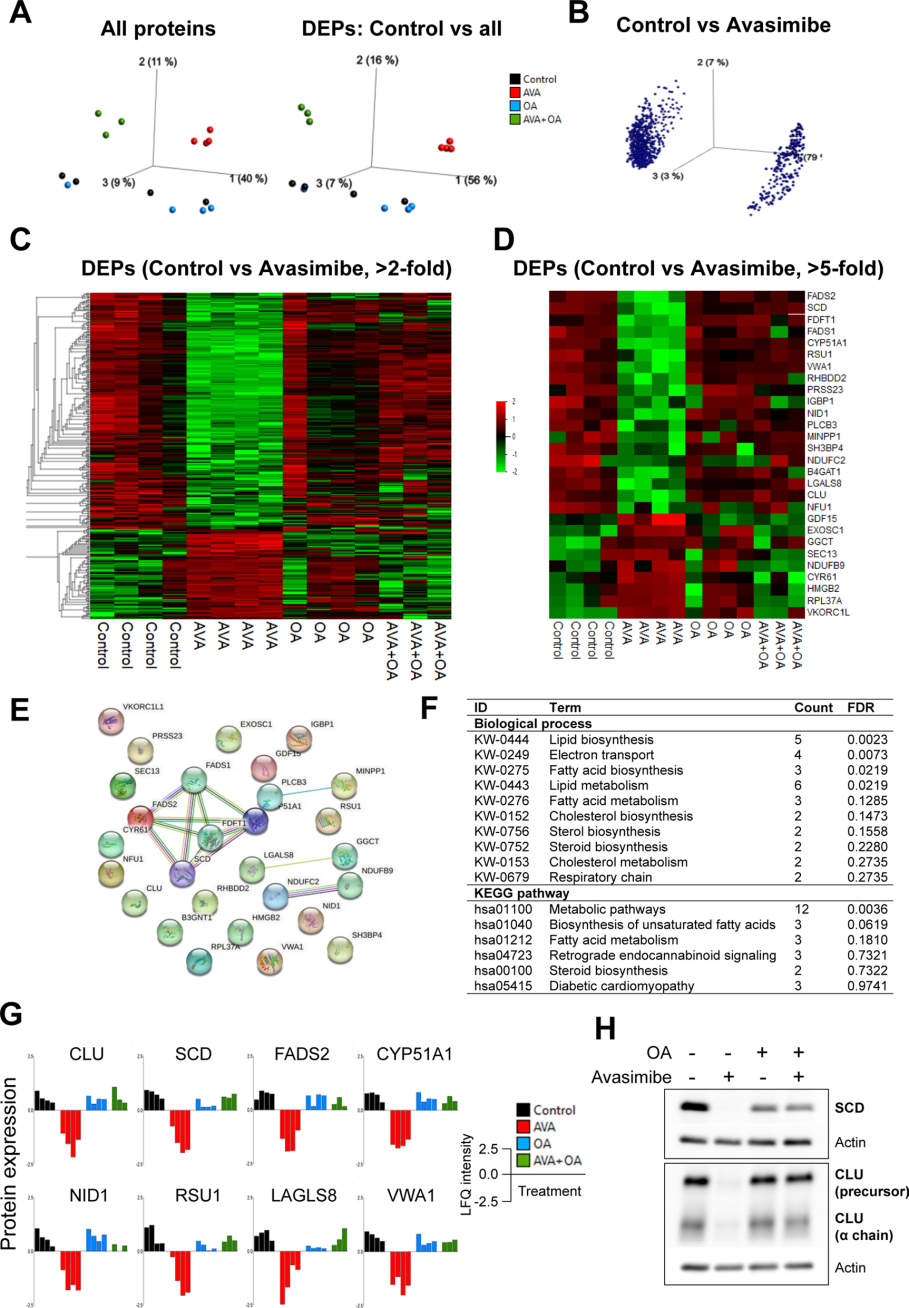
Proteome analysis of PANC-1 cells exposed to avasimibe, with or without oleic acid. Cells treated with avasimibe (AVA; 5 µM), oleic acid (OA; 100 µM) or AVA + OA for 48 h prior to LC–MS/MS. **A**. PCA-sample plot in which each dot represents an individual sample colored according to the treatment group. **B**. PCA-variable plot showing the distribution of differentially expressed proteins (DEPs) between control and AVA-treated samples; each dot represents an individual protein. **C-D.** Heatmaps showing the distribution of all proteins with > twofold (in **C**; n = 289) and > fivefold (in **D**; n = 28) change in expression between AVA-treated and control samples. **E.** STRING network and **F.** Gene ontology enrichment of biological processes and KEGG pathway of DEPs (presented in **D**). **G.** Bar diagrams for selected DEPs (from **D**) with similar expression pattern across all samples from the same treatment group. **H.** Validation of SCD and CLU protein expressions by immunoblotting. *CLU* clusterin, *CYP51A1* cytochrome P450 family 51 subfamily A member 1, *FADS2* fatty acid desaturase 2, *LFQ* label-free quantitation, *LGALS8* Galectin 8, *NID1* Nidogen 1, *RSU1* Ras suppressor protein 1, *SCD* stearoyl-CoA desaturase, VWA1, von Willebrand factor A domain containing 1

Finally, we explored possible mechanisms that may explain the relationship between cholesterol balance, fatty acids, and PDAC cell behavior. As the SOAT1 inhibitor avasimibe induced the most conspicuous changes in PDAC cell behavior, we investigated whole cell proteomes of PANC-1 cells treated with avasimibe, in the presence or absence of OA. Mass spectrometry (MS) analysis identified a total of 34,274 peptides corresponding to total 3261 proteins. The complete MS data is provided in Additional file 3: Table S2. Data visualization was carried out by principal component analysis (PCA; Fig. 6A, B). A 3D-PCA plot for samples (Fig. 6A, left panel) indicated heterogeneity among individual samples from both the control and the OA group, while no apparent difference between the two groups was observed. It further revealed a distinct expression pattern in the avasimibe (AVA) and AVA + OA treated groups compared to the control group, which was confirmed by PCA-sample plot for differentially expressed proteins (DEPs; Fig. 6A, right panel). Overall, 1142 proteins, accounting for ~ 35.1% of all proteins, showed significant change in expression across the four groups (p < 0.05; Additional file 4: Table S3). Comparison of the total proteome between control and AVA-treated groups revealed 990 differentially expressed proteins (DEPs), which account for ~ 30% of all identified proteins (Additional file 4: Table S3). The distribution of these proteins was visualized using a PCA-variable plot, in which each variable corresponds to an individual protein (Fig. 6B). Interestingly, the expression of the majority of these 990 DEPs (~ 78%), was significantly downregulated in the AVA-treated group compared to the control group (Fig. 6C). Of these 990 DEPs, the expression levels of 289, 92, 28 proteins were found altered by > 2-, > 3-, and > fivefold, respectively (Additional file 4: Table S3). Heatmaps for DEPs with > 2- and > fivefold change in expression are shown in Fig. 6C and D, respectively, while the heatmap for DEPs with > threefold change is shown in Additional file 2: Fig. S6. The heatmap in Fig. 6C shows a clear pattern with downregulation of the majority of DEPs (n = 209 i.e., ~ 72.3%) following AVA-treatment compared to control, which appears to be restored upon exposure to OA (AVA + OA group). Enrichment analysis revealed the involvement of these 289 DEPs in a broad range of biological processes, including biosynthesis of lipids (including sterols), metabolism of lipids and fatty acids, apoptosis, and cell adhesion (Additional file 5: Table S4).

Next, we investigated the top 28 DEPs with > fivefold change in their expression levels following AVA treatment compared to controls (Heatmap in Fig. 6D). A STRING network of these proteins is shown in Fig. 6E. These proteins were subsequently grouped according to the biological processes and the KEGG pathways to which they belong (Fig. 6F). A detailed functional annotation all DEPs is provided in Additional file 5: Table S4. The expression pattern for eight DEPs showing a similar expression pattern across all samples, is shown in Fig. 6G. These proteins include CLU, CYP51A1, NID1, RSU1, FADS2, LGALS8, SCD, and VWA1. Each of these proteins showed a significant downregulation following AVA-treatment, which was restored by OA. Functionally, CYP51A1, FADS2, and SCD contribute to lipid biosynthesis, whereas CLU, LGALS8, and RSU1 are involved in regulation of cell growth and apoptosis, and NID1 and VWA1 contribute to interaction with the extracellular matrix. In contrast, comparison of the OA-treated group with the control group identified only 119 DEPs (~ 3.6% of all proteins, Additional file 2: Fig. S7B), of which only 22 showed > twofold change (Additional file 2: Fig. S7C). Most DEPs between the control and the OA groups are involved in the transport of proteins and metabolites, and biosynthesis of lipids and proteins (Additional file 2: Fig. S7D-E; Additional file 5: Table S4). The results presented in Fig. 6G were validated with immunoblotting for two selected proteins, SCD and CLU (Fig. 6H).

## Discussion

**Fig. 7:**
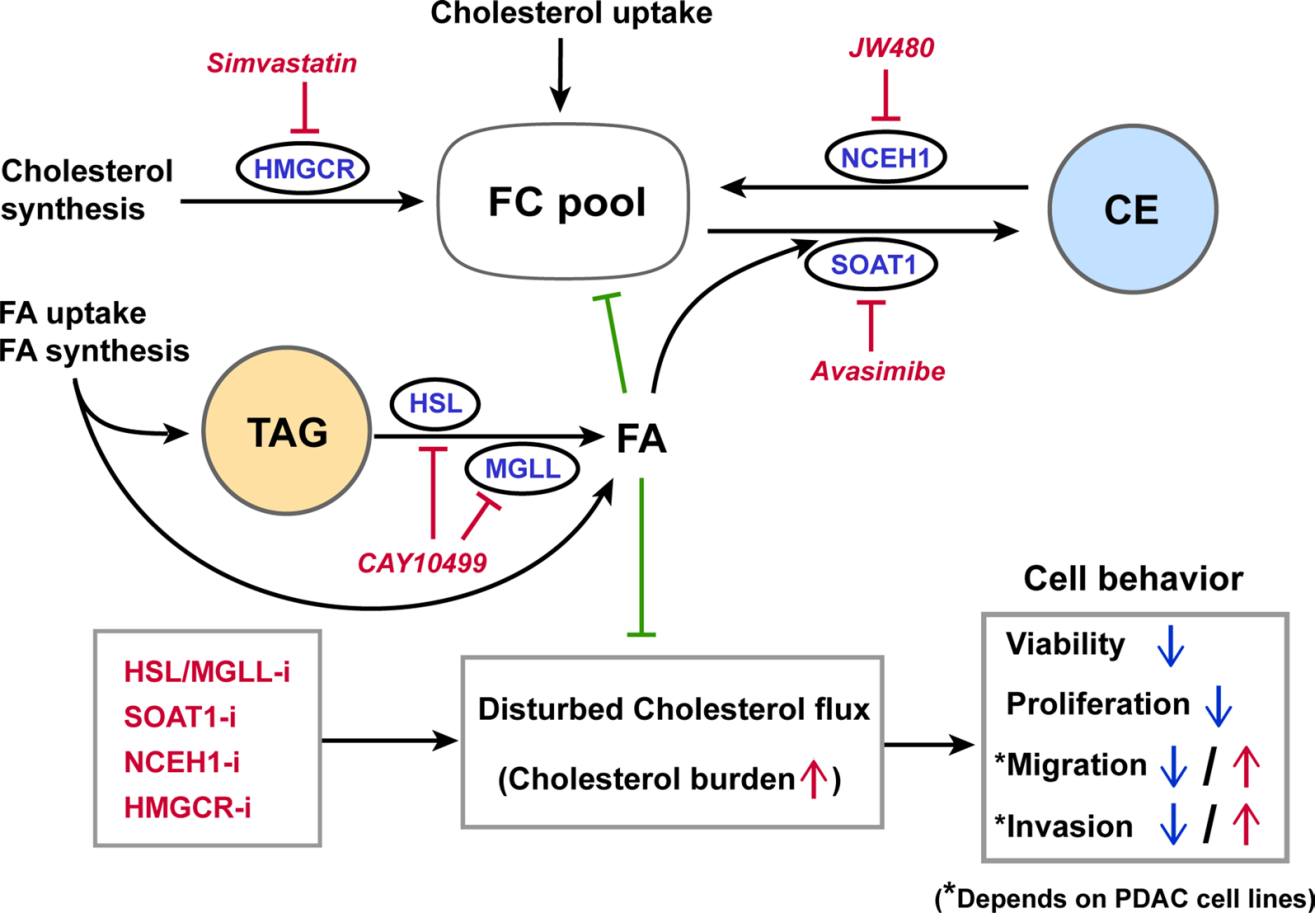
Schematic presentation of how cholesterol flux inhibitors affect cholesterol balance in PDAC cells and the impact of fatty acids. Intracellular cholesterol flux can be blocked at the levels of by inhibition of cholesterol synthesis (HMGCR-i), cholesterol esterification (SOAT1-i), and CE lipolysis (NCEH1-i). In addition, inhibition of HSL/MGLL (HSL/MGLL-i) reduces FA availability. Disturbed cholesterol flux leads to increased cholesterol burden, which alters PDAC cell behavior. FA improves the intracellular cholesterol balance and counteracts the effects of cholesterol flux inhibition in PDAC cells. *CE* cholesteryl ester, *FA* fatty acids, *HMGCR* 3-Hydroxy-3-Methylglutaryl-CoA reductase, *HSL* hormone-sensitive lipase, *MGLL* monoacylglycerol lipase, *NCEH1* neutral cholesterol ester hydrolase 1, *SOAT1* sterol O-acyltransferase 1, *TAG* triacylglycerol

Cholesterol and different components in cholesterol transport and metabolism pathways have become attractive targets for novel diagnostic and therapeutic strategies to treat various cancers, including PDAC [16]. This is not only because intracellular cholesterol flux is so critical for the invasive behavior of cancer cells and cancer progression in general, but also because many cholesterol-regulating drugs, such as HMGCR inhibitors (statins), cholesteryl ester transfer protein (CETP) inhibitors, and niacin derivatives have already been developed and are used clinically for other disorders such as hypercholesterolemia. In this study, we found that PDAC cells have increased levels of cellular FC but reduced ability to store CE. Disturbing cholesterol flux at the levels of cholesterol esterification, CE lipolysis and cholesterol synthesis generally suppressed cell growth and migration in the examined PDAC cells. However, some of the inhibitors of cholesterol flux also promoted cell invasion, depending on cell type and the pathway inhibited, reflecting heterogeneity in cholesterol flux among the different PDAC cell lines. Interestingly, blocking cholesterol flux by SOAT1 inhibition altered the expression of a broad range of proteins in PDAC cells, far beyond components involved exclusively in lipid metabolism. Many of these changes in the PDAC proteome and in cell behavior induced by blocking cholesterol flux were restored by OA, underlining the importance of fatty acids for cellular cholesterol balance in PDAC (Fig. 7).

The expression pattern of proteins involved in lipid flux regulation not only differs between HPDE and PDAC cells, but varies also among different PDAC cell lines, indicating a complex heterogeneity in their lipid metabolism. PDAC cells expressed higher levels of LDLR as well as HMGCR and SQLE compared to HPDE cells, implying their increased cholesterol uptake and synthesis, respectively. This pattern is in accordance with the notion that cancer cells have an increased demand for cholesterol [16]. The level of FC was higher in PDAC cells than in HPDE cells, which is consistent with findings from previous studies [28, 29]. LDs are hubs for intracellular lipid turnover, including fatty acids and cholesterol. FC can be esterified with fatty acids and stored in LDs as CE, while CE can be degraded via lipolysis or lipophagy to release FC [30]. When all cells were grown in the same medium supplemented with 10% FBS, the CE content was lower in PDAC cells compared to HPDE cells. In addition, the expression of SOAT1, the enzyme responsible for CE synthesis, was lower in the three PDAC cell lines than in HPDE cells, consistent with reduced CE storage in PDAC. The increased cholesterol uptake/synthesis and reduced ability for cholesterol esterification may make PDAC cells vulnerable to interventions disturbing intracellular cholesterol flux. However, a study by Li et al. reported that CE content is higher in PDAC tissue than in normal pancreatic tissue, and higher in PDAC cell lines compared to HPDE cells [34]. In their study, PDAC cells were grown in medium supplemented with FBS, while HPDE cells were grown in serum-free medium. As serum is rich in both FA and cholesterol, and we also found that LD content in PDAC and HPDE cells is largely affected by exogenous lipids, this discrepancy might be due to different culture conditions. In addition, it might be that CE storage is higher in normal pancreatic duct epithelial cells than in pancreatic acinar cells, which may explain the discrepancy in relative CE content when comparing CE levels in cultured PDAC cells with HPDE cells, or PDAC tissue with normal pancreatic tissue. As PDAC cells have reduced LD storage and CE content, but elevated intracellular levels of FC, these findings suggest that PDAC cells have a reduced capacity to buffer intracellular lipid flux by incorporating them into LDs. Moreover, it seems that the presence of exogenous cholesterol could prominently increase LD storage (mainly CE), more than the presence of fatty acids alone (mainly TAG), indicating that synthesis and/or turnover of LDs are closely associated with cellular cholesterol turnover in both PDAC and HPDE cells.

In LDs, a continuous turnover of cellular cholesterol takes place through simultaneous esterification and lipolysis. This seemingly futile circle fine tunes the regulation of intracellular cholesterol flux [44, 45]. Except for hepatocytes and small intestine epithelial cells, the main enzyme for intracellular esterification of FC is SOAT1 [46]. SOAT1 plays a key role by storing potentially harmful intracellular FC as less harmful CE into LDs. The effect of SOAT1 inhibition largely depends on the accumulated amount of FC. PDAC cells have a relatively higher cholesterol uptake and synthesis, and a higher FC content than HDPE cells, yet they contain lower levels of SOAT1. This means that the inhibition of the already low SOAT1 in PDAC cells may induce a severe accumulation of FC. This feature (high FC and low SOAT1) makes PDAC cells potentially vulnerable to SOAT1 inhibition. It was recently reported that cholesterol esterification by SOAT1 prevents the negative feedback on cholesterol synthesis elicited by FC in PDAC cells, thereby promoting the mevalonate pathway [38]. This may explain why abrogation of cholesterol esterification either by SOAT1 knockdown or by the SOAT1 inhibitor avasimibe suppresses PDAC growth and metastasis [34]. Moreover, increased CE storage is suspected to contribute to chemotherapy resistance, while SOAT1 inhibitor synergistically suppresses PDAC growth when given together with gemcitabine [47]. In line with these findings, we found that inhibition of SOAT1 by avasimibe prominently increased cell death and inhibited cell proliferation in all the three PDAC cell lines examined and suppressed cell migration in MIA PaCa-2 and PANC-1 cells. However, it seems that SOAT1 inhibition promoted cell migration and invasion in BxPC-3 cells. These findings suggest that the effect of suppressing cholesterol esterification is cell-type dependent, which is in accordance with the considerable genomic and phenotypic heterogeneity that is well established for PDAC cell lines [48]. Whether this is related to effects of oncogenic KRAS mutations (e.g., in MIA PaCa-2 and PANC-1, but not BxPC-3), or connected to other unknown mechanisms, needs further study. SOAT1 inhibition may well increase the intracellular cholesterol burden. This is supported by the observed increase in cellular FC content and aggregation, increased expression of ABCA1, and decreased expression of HMGCR.

Lipolysis of CE are less studied in PDAC. Several candidate enzymes may function as neutral CE esterase, such as HSL [49] and NCEH1 [50], yet it is unclear whether they play a role in cholesterol homeostasis in PDAC. HSL catalyzes lipolysis of a broad range of substrates, including TAG, DAG, MAG, and CE [51]. CAY10499, an inhibitor of HSL/MGLL, has been reported to suppress PDAC cell invasion by reducing the availability of fatty acids that would otherwise fuel cancer metastasis [52]. However, whether HSL inhibition affects cholesterol flux in PDAC has not been investigated previously. We found that CAY10499, but not BAY599435 (an HSL-specific inhibitor), suppressed PDAC. Thus, it is possible that the observed suppressive effect of CAY10499 in PDAC depends on inhibition of MGLL in addition to inhibition of HSL. NCEH1 (also known as KIAA1363) is known to lipolyze CE in macrophages [53] and is overexpressed in various cancers [54]. NCEH1 inhibition has been reported to suppress prostate cancer, but it is unclear if this suppression disturbs cellular cholesterol balance [55–57]. NCEH1 expression levels are inversely correlated with disease-free survival of PDAC patients [58–60]; however, it remains unclear whether NCEH1 regulates cholesterol balance in PDAC. We found that NCEH1 inhibitor JW480 significantly increased CE content in the presence of FA, indicating that NCEH1 might be an important enzyme responsible for CE lipolysis in PDAC. Furthermore, we observed that inhibition of NCEH1 suppressed PDAC cell growth and migration, but apparently increased cell invasion.

HMGCR is a rate-limiting enzyme in the cholesterol synthesis pathway. HMGCR inhibitors such as statins have been widely used clinically for their cholesterol-lowering effect [61]. Statins have been shown to exhibit tumor-suppressive properties in multiple cancer types [62, 63]. However, the effects of statins on PDAC seem to be complex and controversial [36, 39, 64–66]. It has recently been reported that the therapeutic effect of statins on PDAC is dependent on tumor differentiation grade [67]. In our study, inhibition of HMGCR with simvastatin suppressed cell survival and proliferation in all three PDAC cell lines, and inhibited cell migration (except in BxPC-3), and invasion. Although inhibition of HMGCR slightly reduced cellular cholesterol content, it also induced aggregation of FC and increased the expression of ABCA1, indicating disturbed intracellular cholesterol flux.

We found that the impact of inhibitors targeting intracellular cholesterol flux was influenced by the availability of external lipids. Upon inhibition of LD lipolysis (HSL/MGLL and NCEH1), cholesterol esterification (SOAT1) or cholesterol synthesis (HMGCR), we observed decreased HMGCR, increased ABCA1, and reduced PDAC cell survival and proliferation. Most of these alternations could be further aggravated by addition of FC in the culture medium, but surprisingly, these inhibitor-induced changes were completely reversed by addition of OA. OA can activate different intracellular pathways involved in carcinoma cell development [68]. However, reported effects on different tumors seems to be conflicting. For example, OA promotes the growth of highly metastatic tumors [69] while induces cell death in low metastatic tumors [68]. Previously, OA has been reported to inhibit cholesterol synthesis in glioma cells through downregulation of HMGCR expression and activity [70]. In our study, OA slightly reduced cellular FC levels (independent of lipid flux inhibitors) and reduced cholesterol aggregation in PDAC cells, without obvious change in HMGCR expression, indicating that fatty acids may promote intracellular cholesterol balance at multiple levels. Since OA did not significantly increase CE content in the presence of these inhibitors (except for NCEH1 inhibitor), cholesterol esterification may not be the main mechanism by which OA restores the cholesterol balance. As fatty acids can affect cellular membrane components, one possibility would be that they may interfere with the endosomal sorting system and ER membranes [71], and thus promote intracellular cholesterol transport. As discussed above, SOAT1 is a key regulator of cholesterol balance and PDAC cell growth. To our knowledge, avasimibe-induced changes in the PDAC cell proteome have not been reported previously. Noteworthy, the present study demonstrates a substantially altered proteome of PANC-1 cells following the blocking of cholesterol flux by SOAT1 inhibition. The induced changes affected approximately one third of the cell proteome, extending far beyond proteins involved in the cholesterol metabolism per se. The changes mainly involve proteins regulating lipid metabolism, cell survival and growth, as well as interactions with the extracellular matrix. Notably, most of these changes were restored by OA. The proteomics data revealed significantly reduced levels of four lipid metabolism regulators, CLU, SCD, FADS2 and CYP51A1, following avasimibe treatment, however, in the presence of OA there was no change. CLU and SCD, which contribute to lipid transport/cholesterol binding and fatty acid biosynthesis, respectively, were selected for validation of omics data. Immunoblotting of these two proteins confirmed the proteomics data. Interestingly, inhibition of CLU and SCD has been previously shown to suppress proliferation of pancreatic cancer cells and pancreatic tumor growth [72, 73]. SCD is known to be involved in growth and survival of different cancers including breast, lung, liver, colon and pancreas [73, 74]. Moreover, the growth-inhibitory effects induced by pharmacological inhibition of SCD1 in PANC-1 cells [73] and in human colorectal adenocarcinoma LOVO cells (75) were shown to be reduced by OA, in line with the observations in the present study. Overall, these findings indicate that fatty acids regulate cholesterol balance in PDAC cells and thereby may have an impact on PDAC cell behavior.

## Conclusions

The present study shows that PDAC cells have an increased FC content but a reduced ability to store CE. Disturbing the intracellular cholesterol flux through inhibition of cholesterol synthesis and esterification or CE lipolysis generally had a suppressive effect on PDAC cell survival and proliferation, whereas the impact on cell migration and invasion was PDAC cell line dependent. Fatty acids restored PDAC cholesterol balance and abolished most of the effects induced by cholesterol flux disturbance. Hence, while targeting cholesterol flux might be an attractive strategy to suppress PDAC progression, the impact of fatty acids in the tumor microenvironment must be considered.

### Supplementary Information

**Additional file 1: Table S1**. Antibody information.

**Additional file 2: Fig. S1.** Expression of key proteins related to lipid droplet turnover and cholesterol pathway. HPDE and PDAC cells were grown in DMEM supplemented with 10% FBS. Total cell proteins were extracted and analyzed with immunoblotting. **A**. Schematic presentation of lipid turnover through LDs and the key proteins involved. Expression patterns of **B**. major lipases, **C**. major LD-coating proteins and LD-synthesis enzymes, and **D**. proteins involved in cholesterol uptake, synthesis and efflux. ABCA1, ATP binding cassette subfamily A member 1; ATGL, adipose triglyceride lipase; CE, cholesteryl ester; DAG, diacylglycerol; DGATs, Diacylglycerol O-Acyltransferases; FC, free cholesterol; FFA, free fatty acids; HMGCR, 3-hydroxy-3-methylglutaryl-CoA reductase; HSL, hormone-sensitive lipase; LAL, lysosomal acid lipase; LD, lipid droplet; LDLR, Low density lipoprotein receptor; MAG, monoacylglycerol; MGLL, monoglyceride Lipase; NCEH1, neutral cholesterol ester hydrolase 1; SOAT1, sterol O-acyltransferase 1; SQLE, squalene epoxidase; TAG, triacylglycerol. **Fig. S2. **Representative images of cell viability assay (Fig. 2B). A. BxPC-3. **B**. MIA PaCa-2.** C**. PANC-1. BF, bright filed; Hoechst, Hoechst 33342; PI, propidium iodide. **Fig. S3.** The effect of other lipid pathway inhibitors on PDAC cell viability, proliferation and cell density. Cells were incubated in DMEM supplemented with 1% FBS and indicated different enzyme inhibitors (10 µM for all) for 48 hours. **A**. Cell viability was assessed by the percentage of propidium iodide (PI) positive (dead cells) relative to Hoechst 33342 positive (total) nuclei. **B**. Cell proliferation was assessed by BrdU incorporation. **C**. Relative cell density was assessed by crystal violet staining. Results are presented as means ± SD (n = 4-5, *p<0.05 comparing inhibitors and DMSO). **Fig. S4. **Effect of FBS on PDAC cell repression induced by HSL/MGLL, SOAT1 and NCEH1 inhibitors. Cells were incubated in DMEM supplemented with 1% or 10% FBS and indicated different enzyme inhibitors (10 µM for HSL/MGLL-i and NCEH1-i; 5 µM for SOAT1-i) for 48 hours. Relative cell density was assessed by crystal violet staining. Results are presented as means ± SD (n = 5, *p<0.05 comparing inhibitors with DMSO under the same serum concentration; ^#^*p*<0.05 comparing 10% FBS with 1% FBS supplementation). **Fig. S5. **Effect of selected lipid flux inhibitors and oleic acid on cholesterol balance in BxPC-3 cells. Cells were incubated for 48 hours in DMEM supplemented with 1% FBS, with or without the presence of OA (100 µM), FC (50 µM) and various enzyme inhibitors (10 µM for HSL/MGLL-i and NCEH1-i, 5 µM for SOAT1-i and HMGCR-i). **A**. Expression of key proteins involved in cellular cholesterol synthesis and efflux. **B**. Representative images of cells stained with Filipin, indicating cellular distribution of FC. **C**. Quantification of Filipin-positive cell area in **B**. Results are presented as means ± 95% confidence interval in **C** (n = 30-35 images). *p<0.05 comparing inhibitors with DMSO; ^#^*p*<0.05 comparing OA treatment with no OA treatment). FC, free cholesterol; OA, oleic acid. **Fig. S6. **Comparative proteomic analysis of differentially expressed proteins (DEPs) between control and avasimibe-treated PANC-1 cells. Cells were treated with avasimibe (AVA; 5 µM), oleic acid (OA; 100 µM) or AVA+OA for 48 hours prior to mass spectrometry (LC-MS/MS). STRING networks of DEPs with **A.** >2-fold (n=289; *p*<0.05) and **B. **>3-fold (n=92; *p*<0.05) change between control and AVA-treated cells. **C.** Heatmaps showing distribution of all proteins with >3-fold (*p*<0.05) change in expression between control and AVA-treated samples. **Fig. S7. **Comparative proteomic analysis of differentially expressed proteins (DEPs) between control and oleic acid-treated PANC-1 cells. Cells were treated with avasimibe (AVA; 5 µM), oleic acid (OA; 100 µM) or AVA+OA for 48 hours prior to mass spectrometry (LC-MS/MS).** A. **PCA plots showing distribution of samples (PCA-sample plot) and DEPs (PCA-variable plot). Each dot represents an individual sample and individual protein in PCA-sample and -variable plot, respectively. **B-C. **Heatmaps showing distribution of **B.** all DEPs (n=119; *p*<0.05) and **C.** DEPs with >2-fold (n=22; *p*<0.05) change in expression between control and OA-treated samples. **D.** STRING network and **E.** Gene ontology enrichment of biological processes and KEGG pathway of the DEPs presented in **B**.

**Additional file 3: Table S2**. Complete MS/proteome data.

**Additional file 4: Table S3**. MS/proteome data – differentially expressed proteins.

**Additional file 5: Table S4**. GO, KEGG analysis of differentially expressed proteins.

**Additional file 6:** Original TLC and immunoblots.

## Data Availability

The datasets used and/or analyzed during the current study are available from the corresponding author on reasonable requests.

## Abbreviations

ABCA1: ATP binding cassette subfamily A member 1
ATGL: Adipose triglyceride lipase
AVA: Avasimibe
CE: Cholesteryl ester
CLU: Clusterin
DAG: Diacylglycerol
DEPs: Differentially expressed proteins
DGAT-1/2: Diacylglycerol O-acyltransferase 1 and 2
FA: Fatty acids
FC: Free cholesterol
FFA: Free fatty acids
HMGCR: 3-Hydroxy-3-methylglutaryl-coA reductase
HSL: Hormone-sensitive lipase
LAL: Lysosomal acid lipase
LD: Lipid droplet
LDLR: Low density lipoprotein receptor
MAG: Monoacylglycerol
MGLL: Monoacylglycerol lipase
NCEH1: Neutral cholesterol ester hydrolase 1
OA: Oleic acid
PCA: Principal component analysis
PDAC: Pancreatic ductal adenocarcinoma
PL: Phospholipids
SCD: Stearoyl-CoA desaturase
SOAT1: Sterol O-acyltransferase 1
TAG: Triacylglycerol
TLC: Thin-layer chromatography
SQLE: Squalene epoxidase
