# Supplementary material for: Fatty acids abrogate the growth-suppressive effects induced by inhibition of cholesterol flux in pancreatic cancer cells
Source: Cancer Cell Int. 2023 Nov 17;23:276. doi: 10.1186/s12935-023-03138-8 (PMC10657020; doi:10.1186/s12935-023-03138-8)
Supplement: Supplementary file 1 — Additional file 1: Table S1. Antibody information. [file 12935_2023_3138_MOESM1_ESM.docx]

**Additional file 1**

**Table S1. Antibody information**

| **Antigen** | **Cat. #** | **Provider** | **RRID** | |
| --- | --- | --- | --- | --- |
| ABCA1 | NB400-105 | Novus Biologicals | AB_10000630 | |
| ACTIN | sc-1616 | Santa Cruz Biotechnology | AB_630836 | |
| ATGL | #2138 | Cell Signaling Technology | AB_2167955 | |
| CLU | #42143 | Cell Signaling Technology | AB_2799215 |  |
| DGAT1 | 11561-1-AP | Proteintech | AB_2877779 | |
| HMGCR | ab242315 | Abcam | AB_2928124 | |
| HSL (LIPE) | 4107S | Cell Signaling Technology | AB_2296900 | |
| LAL (LIPA) | 12956-1-AP | Proteintech | AB_2078477 | |
| LDLR | 10785-1-AP | Proteintech | AB_2281164 | |
| MGLL | 14986-1-AP | Proteintech | AB_2143189 | |
| NCEH1 | SAB4301148 | Sigma-Aldrich | not available | |
| Plin3 (TIP47) | PA5-82248 | Thermo Fisher Scientific | AB_2789408 | |
| SCD1 | #2438 | Cell Signaling Technology | AB_823634 |  |
| SOAT1 | #35695 | Cell Signaling Technology | AB_2799083 | |
| SQLE | #40659 | Cell Signaling Technology | not available | |
| Goat Anti-Rabbit IgG | 170-6515 | Bio-Rad Laboratories | AB_11125142 | |
| Goat Anti-Mouse IgG | 170-6516 | Bio-Rad Laboratories | AB_11125547 | |
| Rabbit anti-Goat IgG | # 81-1620 | Thermo Fisher Scientific | AB_2534006 | |

ABCA1, ATP binding cassette subfamily A member 1; ATGL, adipose triglyceride lipase; CLU, Clusterin; DGAT1, Diacylglycerol O-Acyltransferase 1; HMGCR, 3-hydroxy-3-methylglutaryl-CoA reductase; HSL, hormone-sensitive lipase; LAL, lysosomal acid lipase; LDLR, Low density lipoprotein receptor; MGLL, monoglyceride Lipase; NCEH1, neutral cholesterol ester hydrolase 1; SCD1, Stearoyl-CoA desaturase 1; SOAT1, sterol O-acyltransferase 1; SQLE, squalene epoxidase.
