# Supplementary material for: Fatty acids abrogate the growth-suppressive effects induced by inhibition of cholesterol flux in pancreatic cancer cells
Source: Cancer Cell Int. 2023 Nov 17;23:276. doi: 10.1186/s12935-023-03138-8 (PMC10657020; doi:10.1186/s12935-023-03138-8)
Supplement: Supplementary file 2 — Additional file 2: Fig. S1. Expression of key proteins related to lipid droplet turnover and cholesterol pathway. HPDE and PDAC cells were grown in DMEM supplemented with 10% FBS. Total cell proteins were extracted and analyzed with immunoblotting. A. Schematic presentation of lipid turnover through LDs and the key proteins involved. Expression patterns of B. major lipases, C. major LD-coating proteins and LD-synthesis enzymes, and D. proteins involved in cholesterol uptake, synthesis and efflux. ABCA1, ATP binding cassette subfamily A member 1; ATGL, adipose triglyceride lipase; CE, cholesteryl ester; DAG, diacylglycerol; DGATs, Diacylglycerol O-Acyltransferases; FC, free cholesterol; FFA, free fatty acids; HMGCR, 3-hydroxy-3-methylglutaryl-CoA reductase; HSL, hormone-sensitive lipase; LAL, lysosomal acid lipase; LD, lipid droplet; LDLR, Low density lipoprotein receptor; MAG, monoacylglycerol; MGLL, monoglyceride Lipase; NCEH1, neutral cholesterol ester hydrolase 1; SOAT1, sterol O-acyltransferase 1; SQLE, squalene epoxidase; TAG, triacylglycerol. Fig. S2. Representative images of cell viability assay (Fig. 2B). A. BxPC-3. B. MIA PaCa-2. C. PANC-1. BF, bright filed; Hoechst, Hoechst 33342; PI, propidium iodide. Fig. S3. The effect of other lipid pathway inhibitors on PDAC cell viability, proliferation and cell density. Cells were incubated in DMEM supplemented with 1% FBS and indicated different enzyme inhibitors (10 µM for all) for 48 hours. A. Cell viability was assessed by the percentage of propidium iodide (PI) positive (dead cells) relative to Hoechst 33342 positive (total) nuclei. B. Cell proliferation was assessed by BrdU incorporation. C. Relative cell density was assessed by crystal violet staining. Results are presented as means ± SD (n = 4-5, *p<0.05 comparing inhibitors and DMSO). Fig. S4. Effect of FBS on PDAC cell repression induced by HSL/MGLL, SOAT1 and NCEH1 inhibitors. Cells were incubated in DMEM supplemented with 1% or 10% FBS and indicated diff [file 12935_2023_3138_MOESM2_ESM.docx]

**Additional file 2: Figures S1-S7**

**
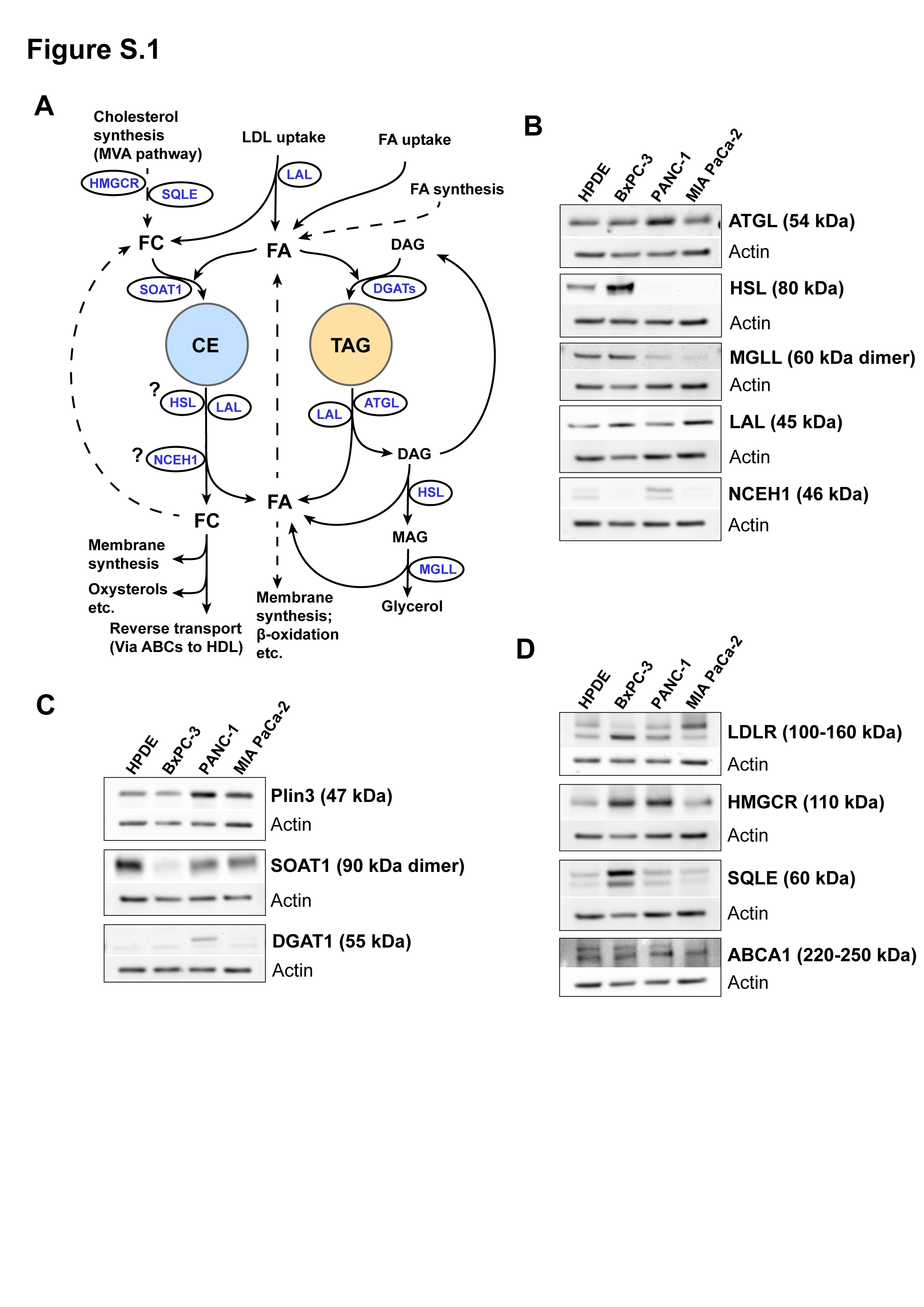
**

**Fig. S1. Expression of key proteins related to lipid droplet turnover and cholesterol pathway.** HPDE and PDAC cells were grown in DMEM supplemented with 10% FBS. Total cell proteins were extracted and analyzed with immunoblotting. **A**. Schematic presentation of lipid turnover through LDs and the key proteins involved. Expression patterns of **B**. major lipases, **C**. major LD-coating proteins and LD-synthesis enzymes, and **D**. proteins involved in cholesterol uptake, synthesis and efflux. ABCA1, ATP binding cassette subfamily A member 1; ATGL, adipose triglyceride lipase; CE, cholesteryl ester; DAG, diacylglycerol; DGATs, Diacylglycerol O-Acyltransferases; FC, free cholesterol; FFA, free fatty acids; HMGCR, 3-hydroxy-3-methylglutaryl-CoA reductase; HSL, hormone-sensitive lipase; LAL, lysosomal acid lipase; LD, lipid droplet; LDLR, Low density lipoprotein receptor; MAG, monoacylglycerol; MGLL, monoglyceride Lipase; NCEH1, neutral cholesterol ester hydrolase 1; SOAT1, sterol O-acyltransferase 1; SQLE, squalene epoxidase; TAG, triacylglycerol.

**
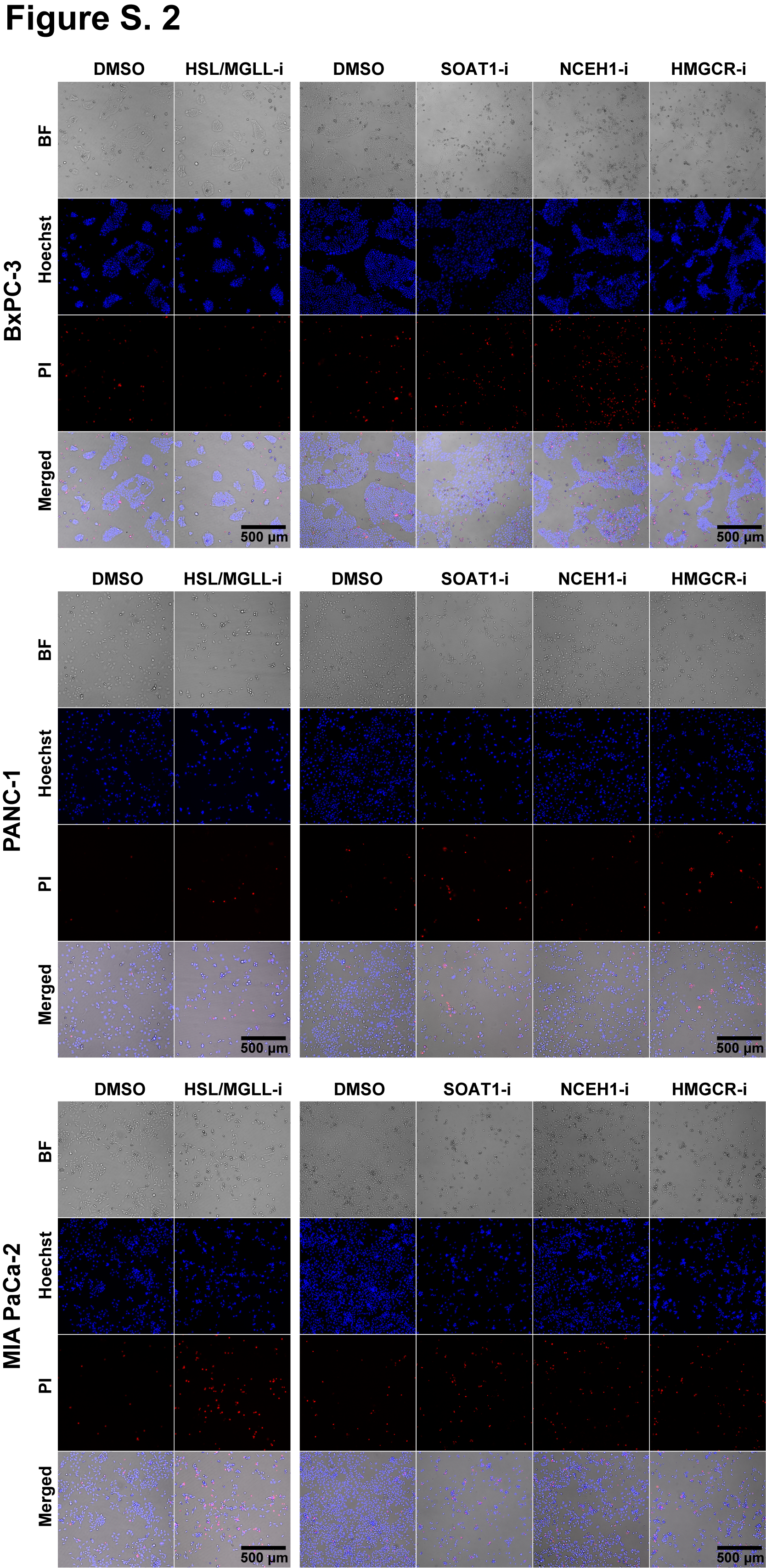
**

**Fig. S2. Representative images of cell viability assay (Fig. 2B). A**. BxPC-3. **B**. MIA PaCa-2. **C**. PANC-1. BF, bright filed; Hoechst, Hoechst 33342; PI, propidium iodide.

**
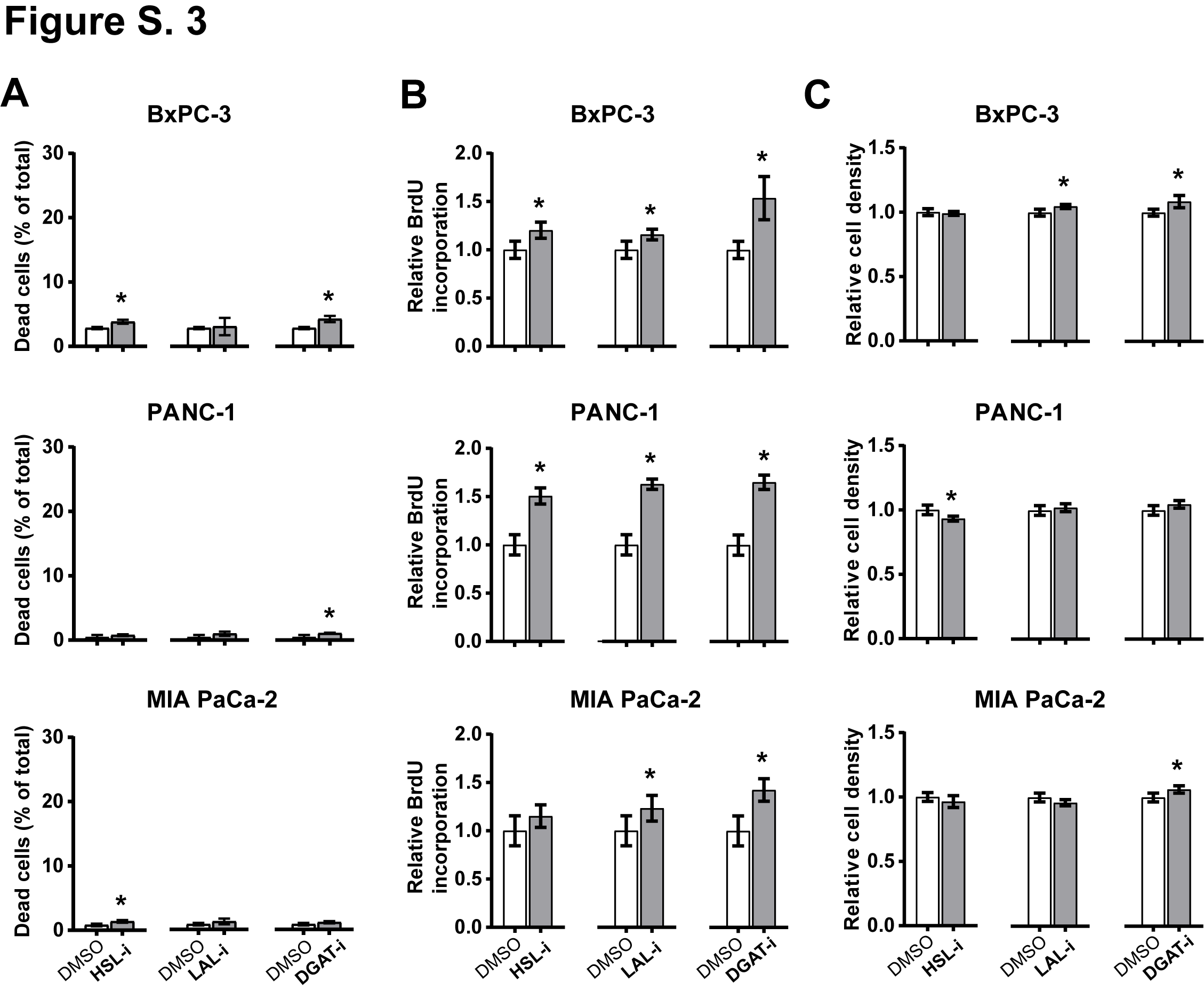
**

**Fig. S3. The effect of other lipid pathway inhibitors on PDAC cell viability, proliferation and cell density.** Cells were incubated in DMEM supplemented with 1% FBS and indicated different enzyme inhibitors (10 µM for all) for 48 hours. **A**. Cell viability was assessed by the percentage of propidium iodide (PI) positive (dead cells) relative to Hoechst 33342 positive (total) nuclei. **B**. Cell proliferation was assessed by BrdU incorporation. **C**. Relative cell density was assessed by crystal violet staining. Results are presented as means ± SD (n = 4-5, **p*<0.05 comparing inhibitors and DMSO).


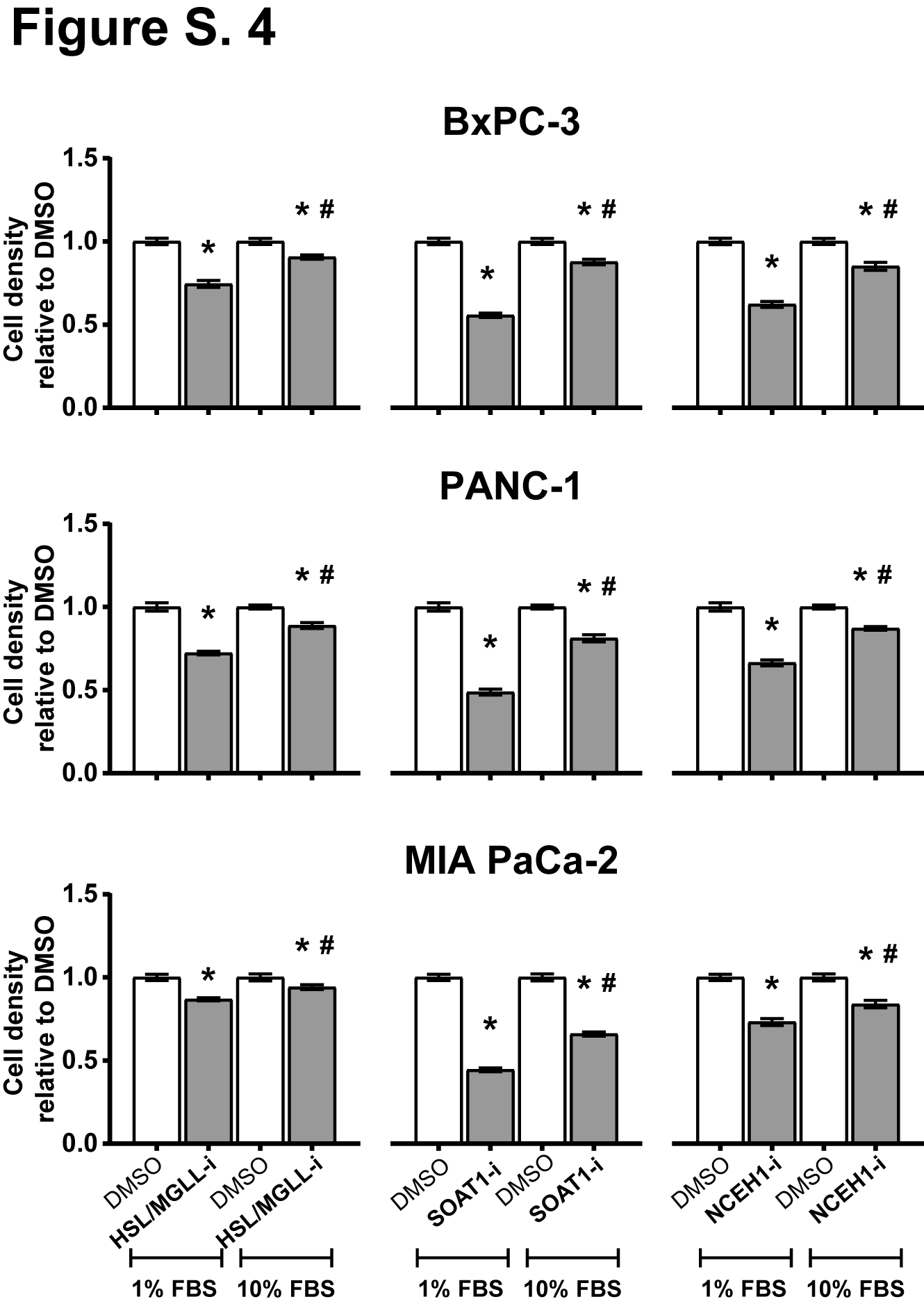


**Fig. S4. Effect of FBS on PDAC cell repression induced by HSL/MGLL, SOAT1 and NCEH1 inhibitors.** Cells were incubated in DMEM supplemented with 1% or 10% FBS and indicated different enzyme inhibitors (10 µM for HSL/MGLL-i and NCEH1-i; 5 µM for SOAT1-i) for 48 hours. Relative cell density was assessed by crystal violet staining. Results are presented as means ± SD (n = 5, **p*<0.05 comparing inhibitors with DMSO under the same serum concentration; ^#^*p*<0.05 comparing 10% FBS with 1% FBS supplementation).


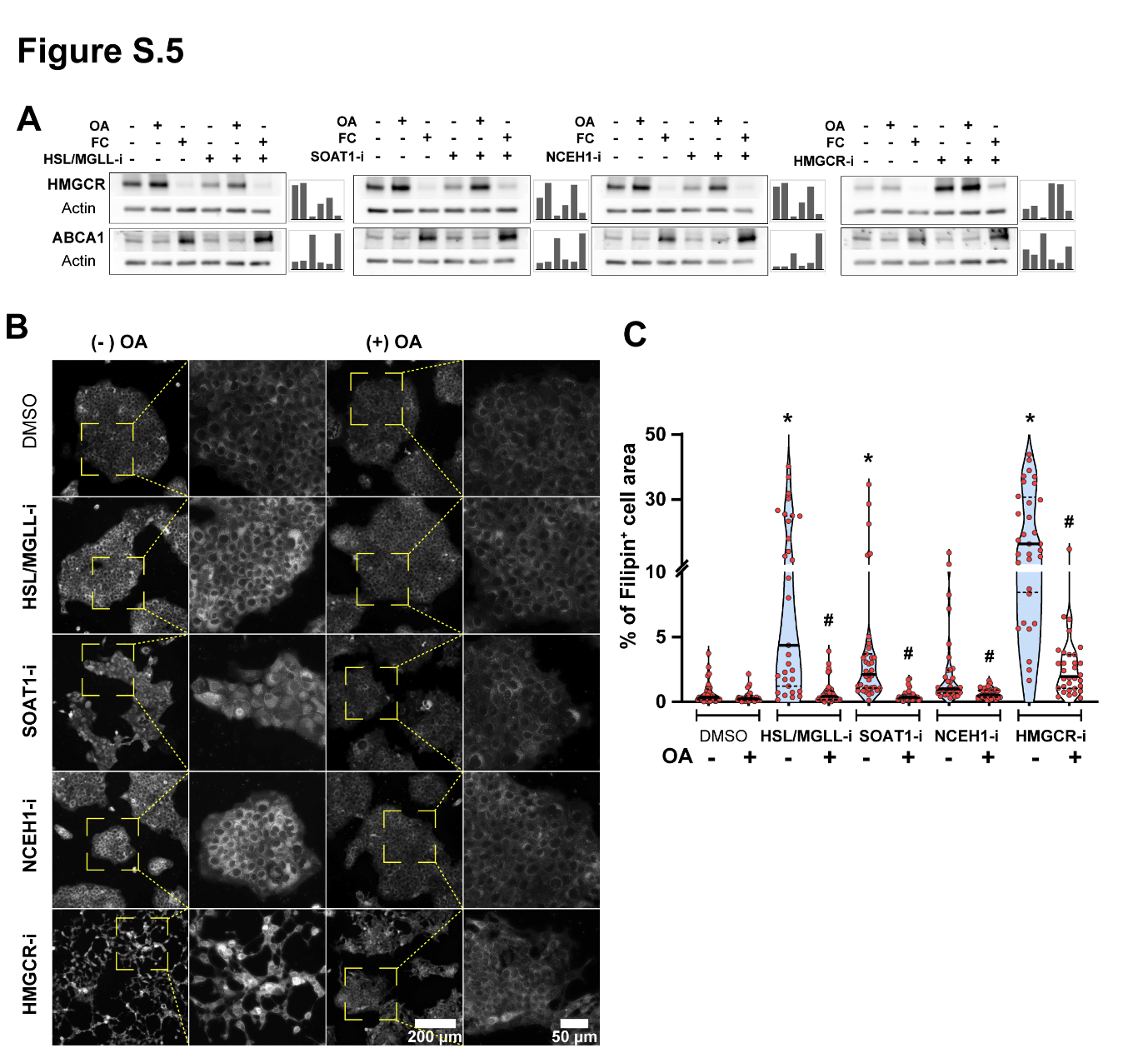


**Fig. S5. Effect of selected lipid flux inhibitors and oleic acid on cholesterol balance in BxPC-3 cells.** Cells were incubated for 48 hours in DMEM supplemented with 1% FBS, with or without the presence of OA (100 µM), FC (50 µM) and various enzyme inhibitors (10 µM for HSL/MGLL-i and NCEH1-i, 5 µM for SOAT1-i and HMGCR-i). **A**. Expression of key proteins involved in cellular cholesterol synthesis and efflux. **B**. Representative images of cells stained with Filipin, indicating cellular distribution of FC. **C**. Quantification of Filipin-positive cell area in **B**. Results are presented as means ± 95% confidence interval in **C** (n = 30-35 images). **p*<0.05 comparing inhibitors with DMSO; ^#^*p*<0.05 comparing OA treatment with no OA treatment). FC, free cholesterol; OA, oleic acid.

**
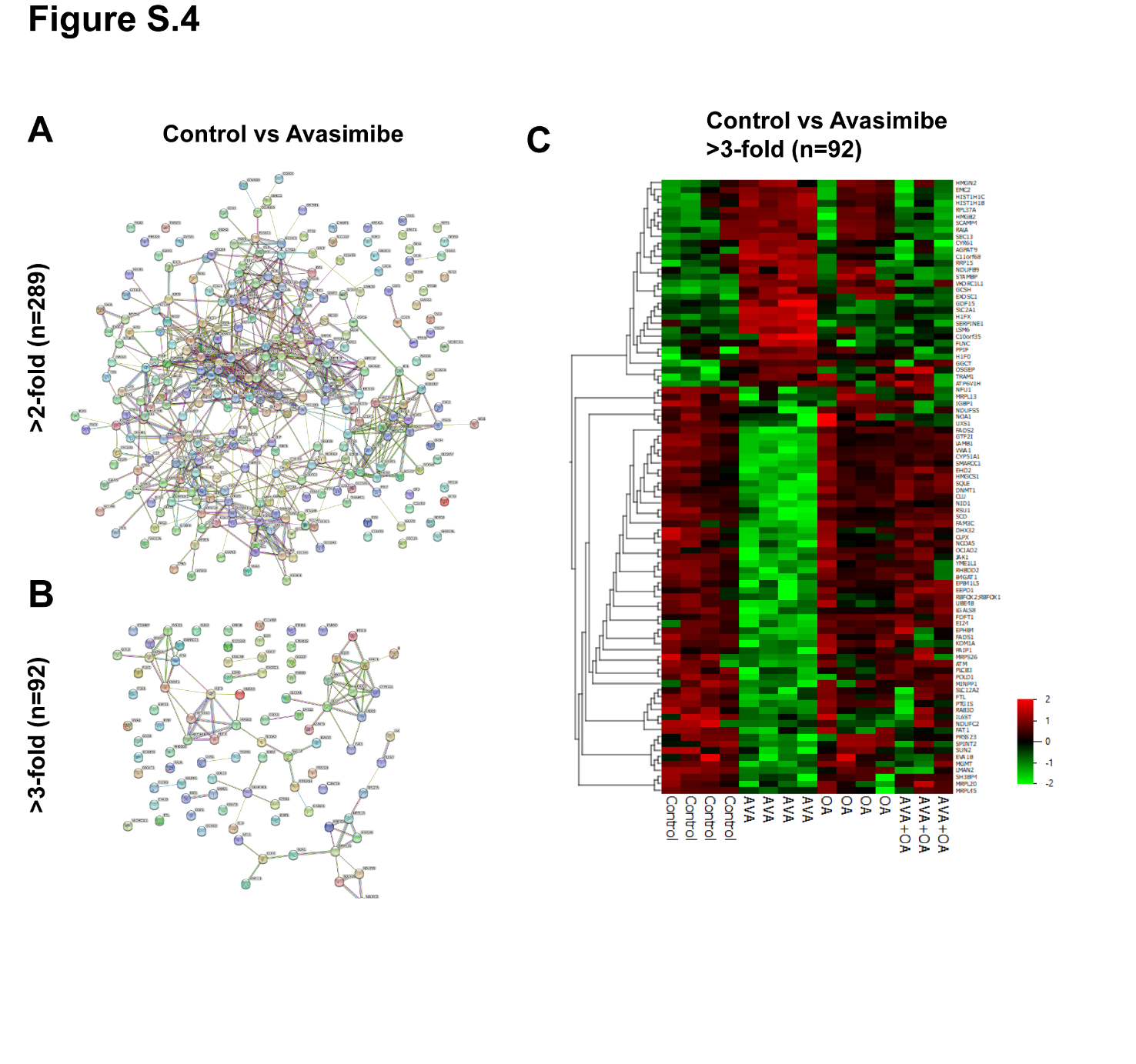
**

**Fig. S6. Comparative proteomic analysis of differentially expressed proteins (DEPs) between control and avasimibe-treated PANC-1 cells.** Cells were treated with avasimibe (AVA; 5 µM), oleic acid (OA; 100 µM) or AVA+OA for 48 hours prior to mass spectrometry (LC-MS/MS). STRING networks of DEPs with **A.** >2-fold (n=289; *p*<0.05) and **B.** >3-fold (n=92; *p*<0.05) change between control and AVA-treated cells. **C.** Heatmaps showing distribution of all proteins with >3-fold (*p*<0.05) change in expression between control and AVA-treated samples.


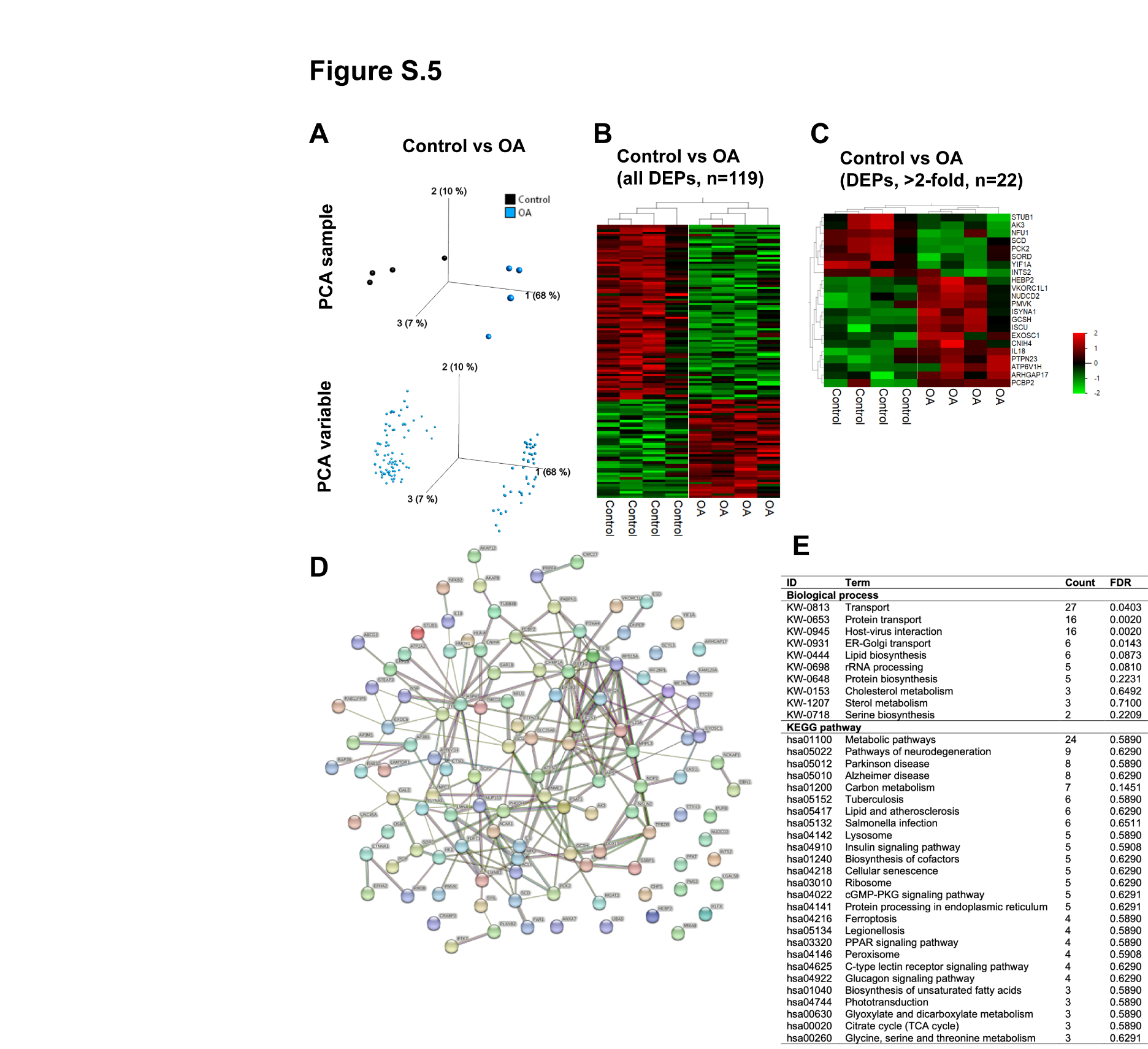


**Fig. S7. Comparative proteomic analysis of differentially expressed proteins (DEPs) between control and oleic acid-treated PANC-1 cells.** Cells were treated with avasimibe (AVA; 5 µM), oleic acid (OA; 100 µM) or AVA+OA for 48 hours prior to mass spectrometry (LC-MS/MS). **A.** PCA plots showing distribution of samples (PCA-sample plot) and DEPs (PCA-variable plot). Each dot represents an individual sample and individual protein in PCA-sample and -variable plot, respectively. **B-C.** Heatmaps showing distribution of **B.** all DEPs (n=119; *p*<0.05) and **C.** DEPs with >2-fold (n=22; *p*<0.05) change in expression between control and OA-treated samples. **D.** STRING network and **E.** Gene ontology enrichment of biological processes and KEGG pathway of the DEPs presented in **B**.
