# Supplementary material for: Fatty acids abrogate the growth-suppressive effects induced by inhibition of cholesterol flux in pancreatic cancer cells
Source: Cancer Cell Int. 2023 Nov 17;23:276. doi: 10.1186/s12935-023-03138-8 (PMC10657020; doi:10.1186/s12935-023-03138-8)

**Additional file 6: original TLC and immunoblots**

**Additional TLC in Figure 1A**

**
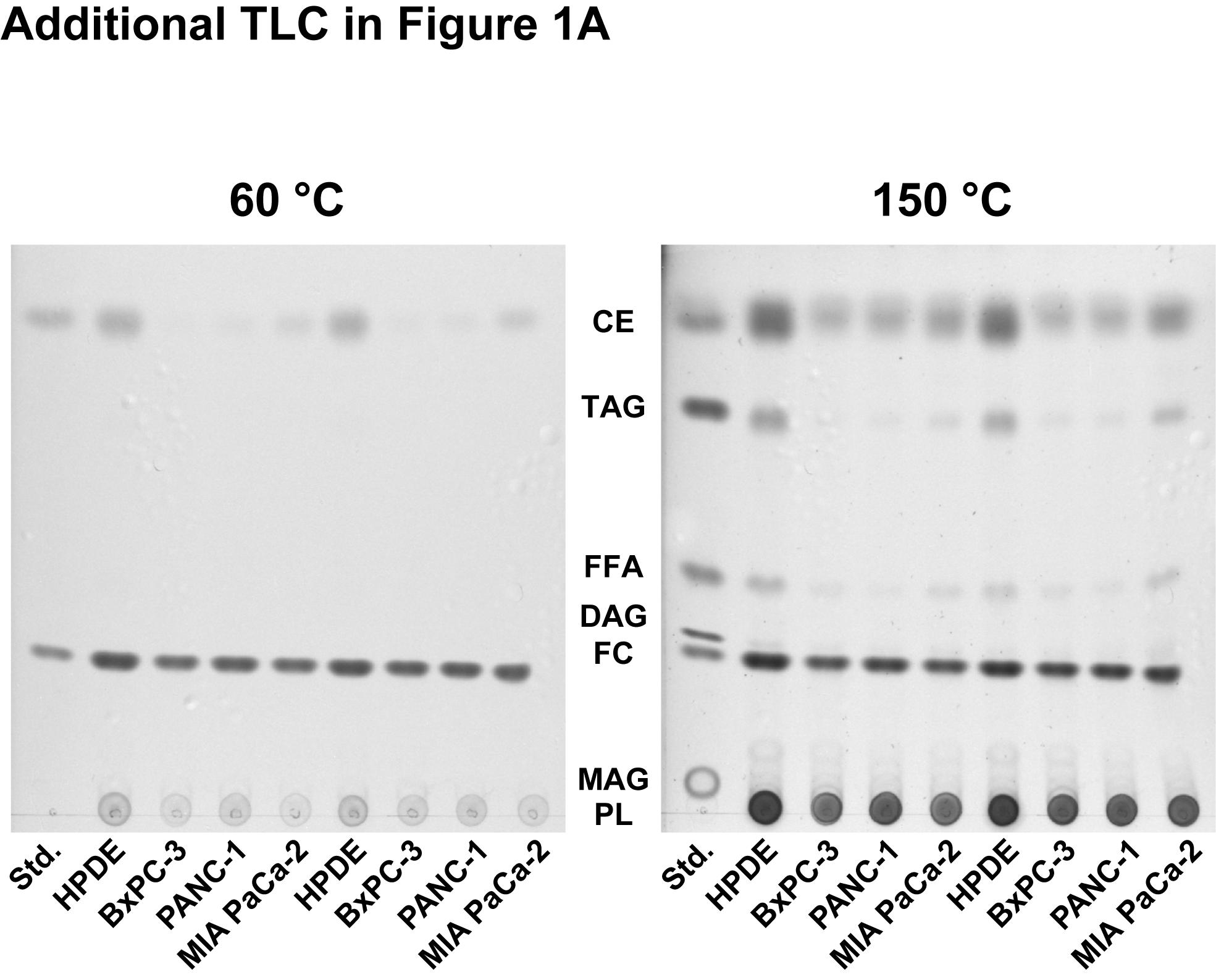
**

**Full blots in Figure S1**


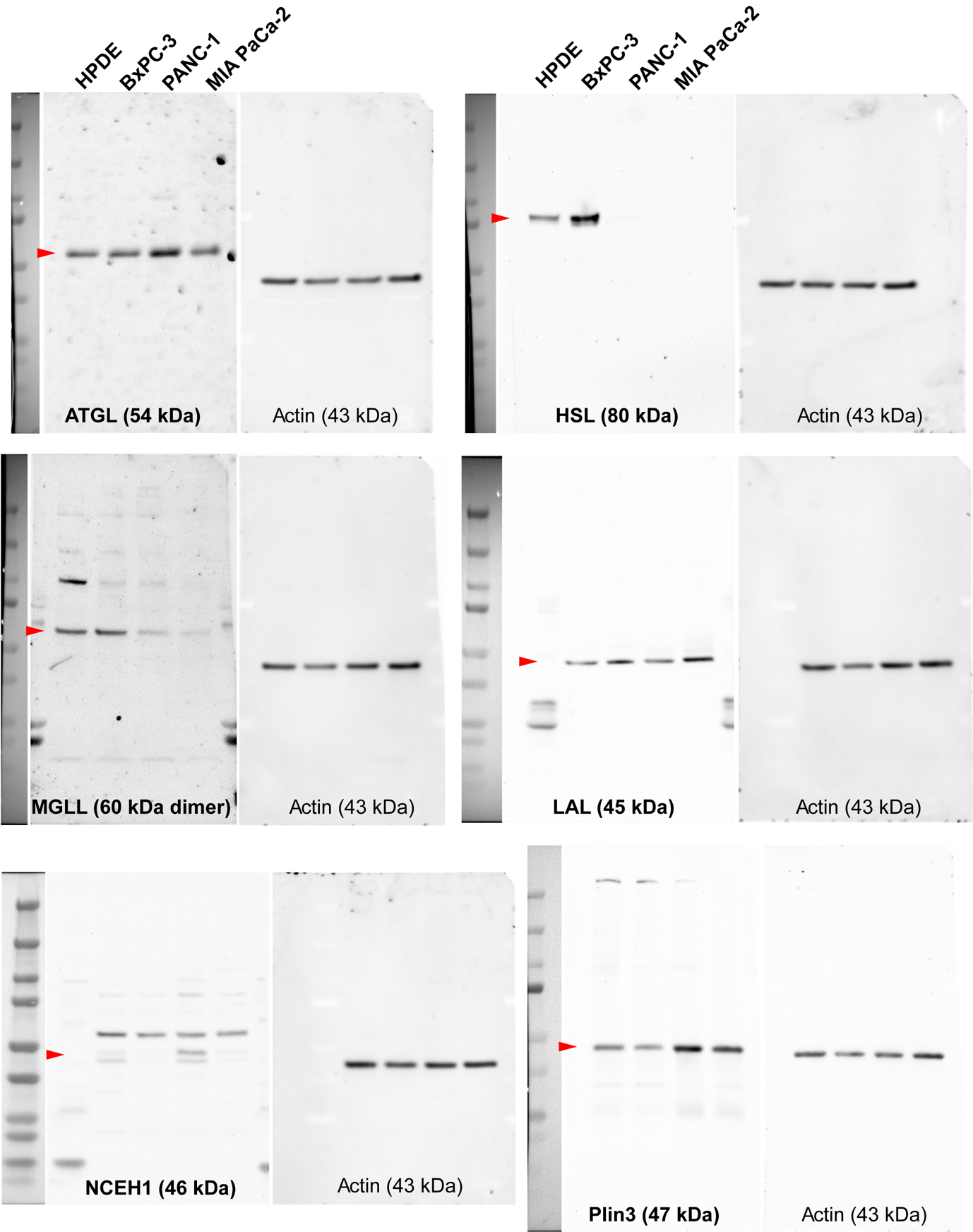


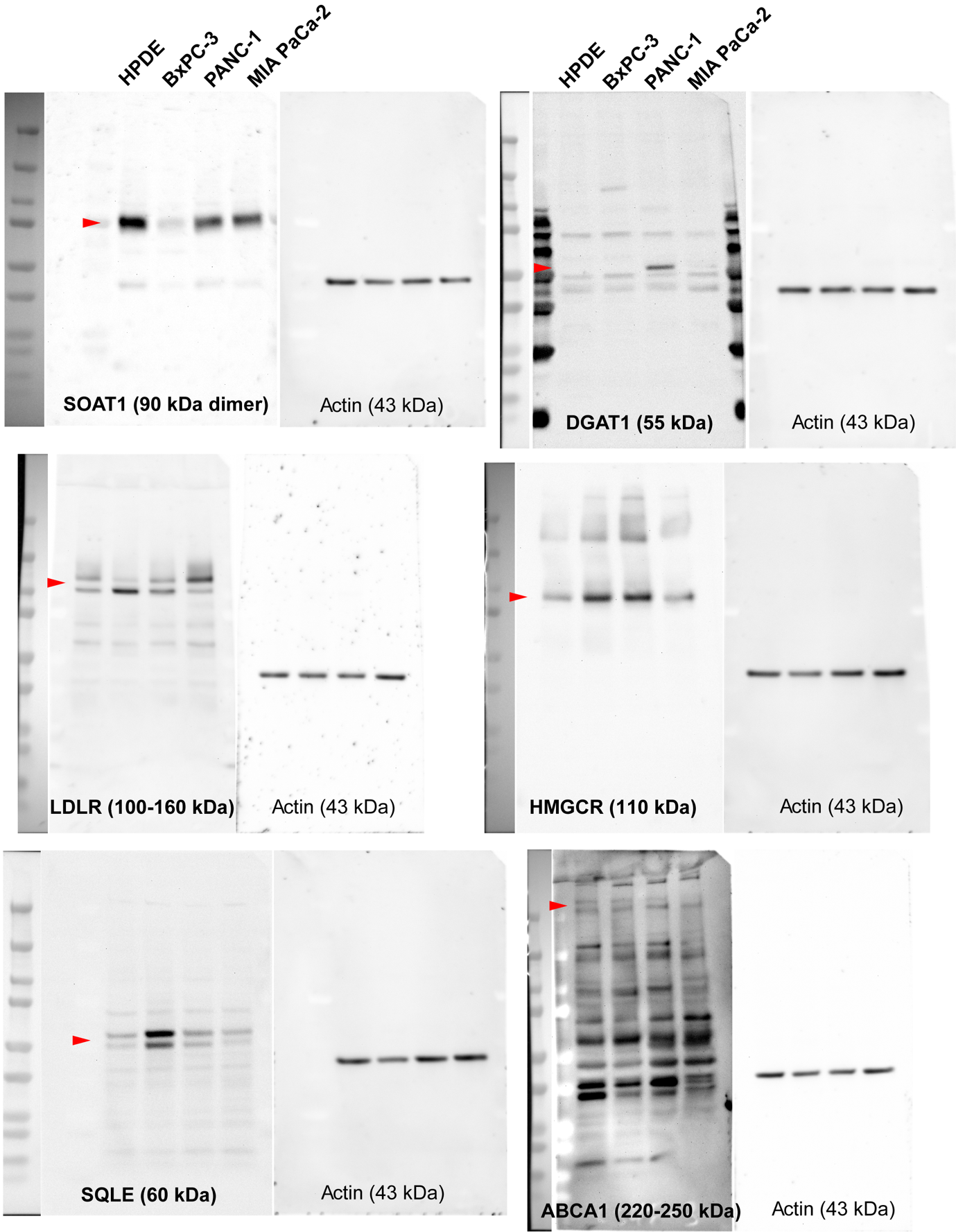


**Full blots in Figure 5A**


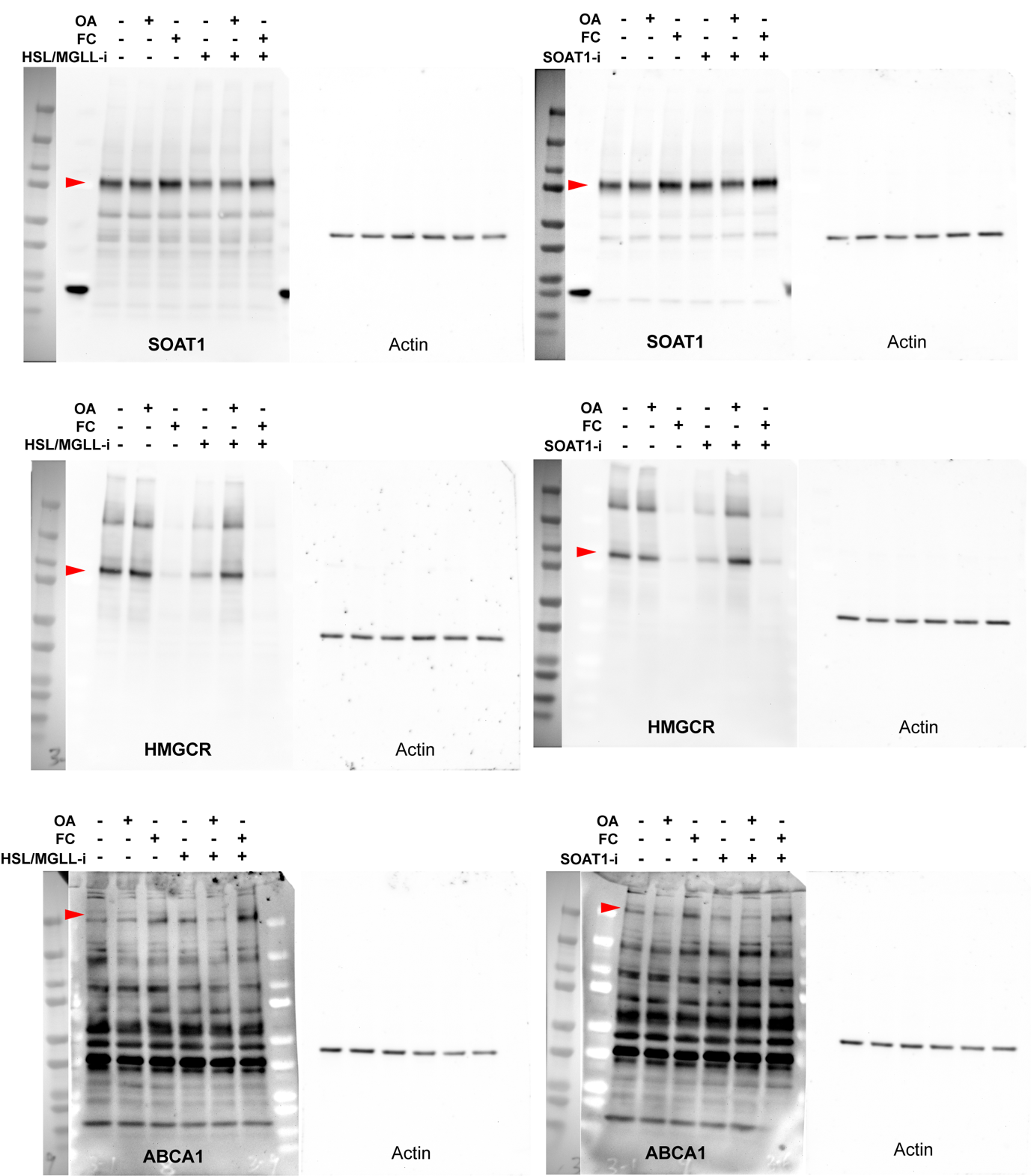


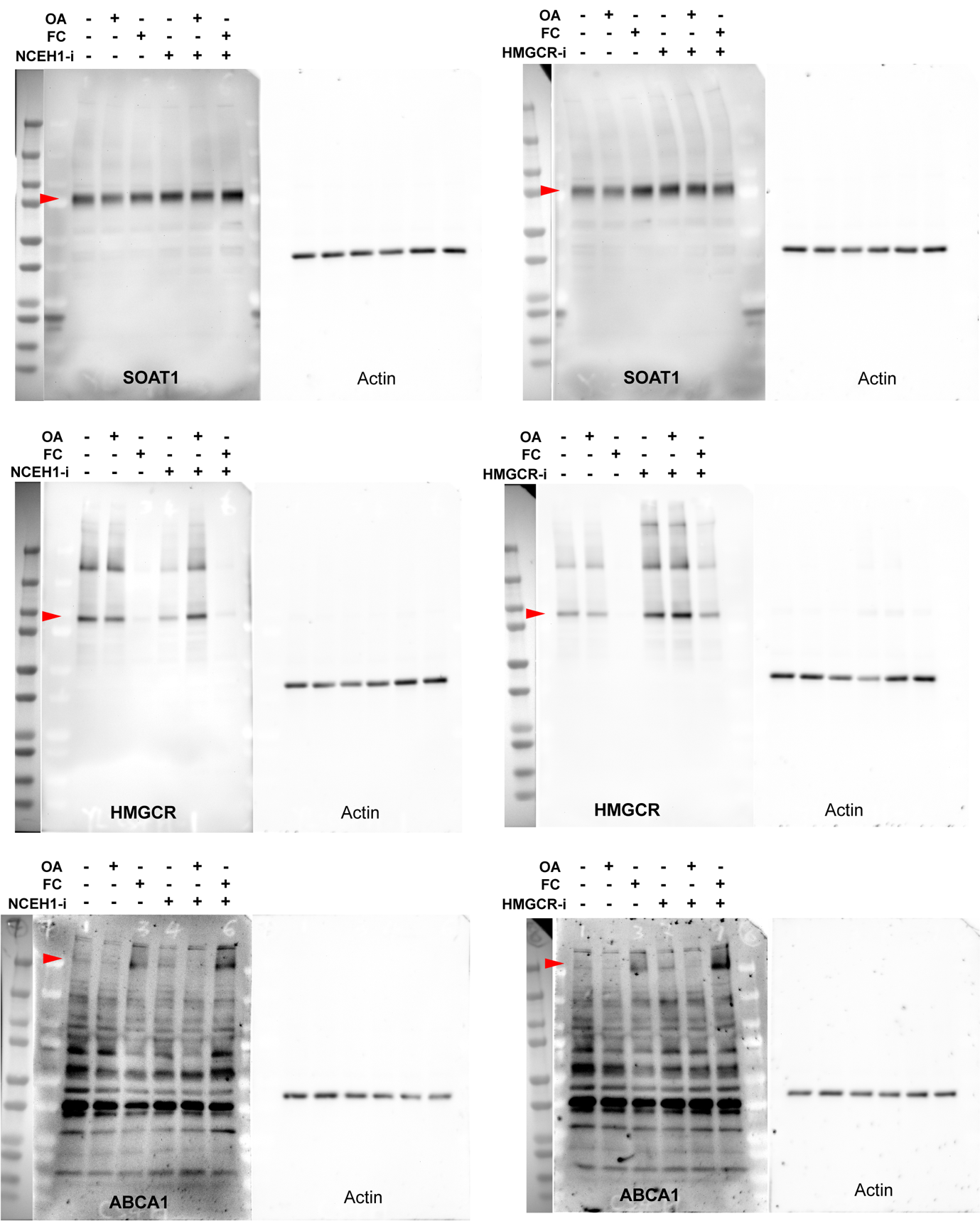


**Full blots in Figure S5A**


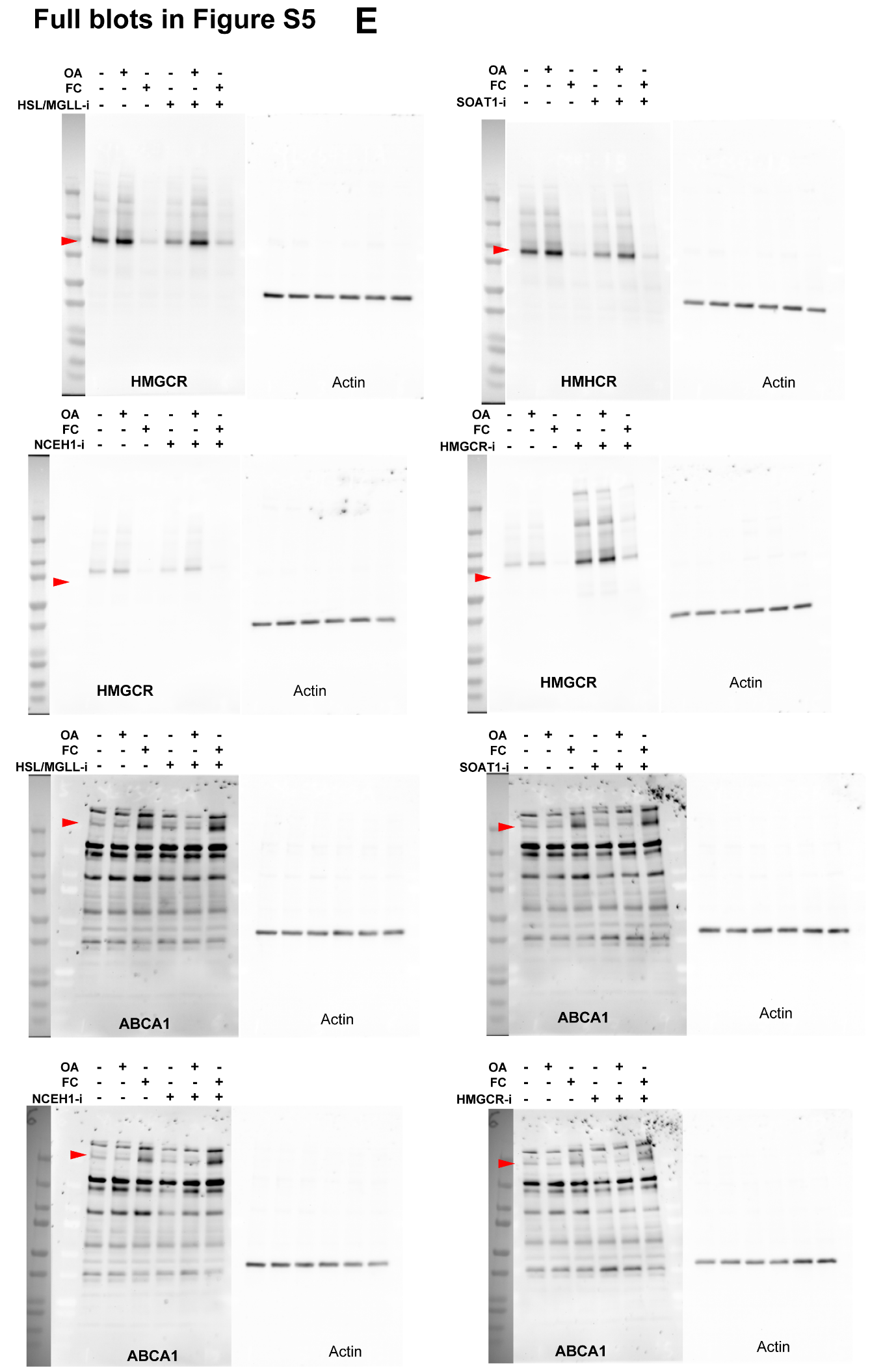


**Full blots in Figure 6H**


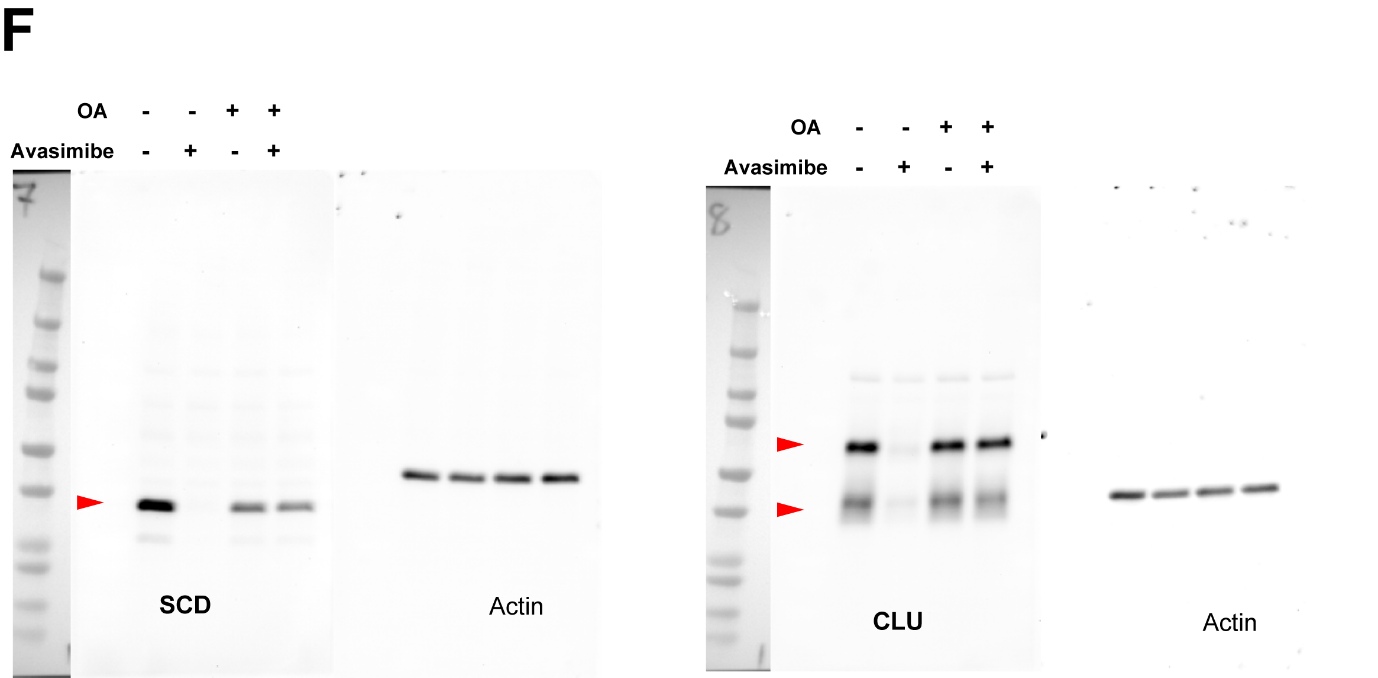

Supplement: Supplementary file 6 — Additional file 6: Original TLC and immunoblots. [file 12935_2023_3138_MOESM6_ESM.docx]
